# Potassium–chloride cotransporter 2 activity dampens induced ictal‐like activity in neocortical slices containing the seizure propagation zone of temporal lobe epilepsy patients

**DOI:** 10.1111/epi.18630

**Published:** 2025-09-11

**Authors:** Alice Falck, Janna Lehnhoff, Mahraz Behbood, Egor Byvaltcev, Annette Aigner, Helena Radbruch, Pawel Fidzinski, Jan‐Hendrik Schleimer, Gabriel M. S. Janach, Noah Döhne, Julia Onken, Thilo Kalbhenn, Thomas Sauvigny, Rudolf A. Deisz, Susanne Schreiber, Martin Holtkamp, Ulf Strauss

**Affiliations:** ^1^ Center for Anatomy Institute for Cell Biology and Neurobiology, Charité–Universitätsmedizin Berlin, corporate member of Freie Universität Berlin and Humboldt‐Universität zu Berlin Berlin Germany; ^2^ Department of Neurology With Experimental Neurology Charité–Universitätsmedizin Berlin, corporate member of Freie Universität Berlin and Humboldt‐Universität zu Berlin Berlin Germany; ^3^ Department of Biology, Institute for Theoretical Biology Humboldt‐Universität zu Berlin Berlin Germany; ^4^ Bernstein Center for Computational Neuroscience Berlin Germany; ^5^ Institute of Biometry and Clinical Epidemiology, Charité–Universitätsmedizin Berlin, corporate member of Freie Universität Berlin and Humboldt‐Universität zu Berlin Berlin Germany; ^6^ Center for Stroke Research Berlin, Charité–Universitätsmedizin Berlin, corporate member of Freie Universität Berlin and Humboldt‐Universität zu Berlin Berlin Germany; ^7^ Department of Neuropathology Charité–Universitätsmedizin Berlin, corporate member of Freie Universität Berlin and Humboldt‐Universität Zu Berlin Berlin Germany; ^8^ Neuroscience Clinical Research Center, Berlin Institute of Health, Charité–Universitätsmedizin Berlin, corporate member of Freie Universität Berlin and Humboldt‐Universität zu Berlin Berlin Germany; ^9^ Department of Neurosurgery Charité–Universitätsmedizin Berlin, corporate member of Freie Universität Berlin and Humboldt‐Universität zu Berlin Berlin Germany; ^10^ Department of Neurosurgery Evangelisches Klinikum Bethel, Universitätsklinikum OWL der Universität Bielefeld Bielefeld Germany; ^11^ Department of Neurosurgery Klinik und Poliklinik für Neurochirurgie, Universitätsklinikum Hamburg Eppendorf Hamburg Germany; ^12^ Epilepsy Center Berlin‐Brandenburg, Institute for Diagnostics of Epilepsy, Evangelisches Krankenhaus Königin Elisabeth Herzberge Berlin Germany

**Keywords:** chloride homeostasis, KCC2, layer 2/3, microelectrode Array, seizure propagation

## Abstract

**Objective:**

The K^+^/Cl^−^ cotransporter (KCC2), which acts as the main Cl^−^ extruder in the adult brain, coregulates the driving force and therewith indirectly the amount and polarity of γ‐aminobutyric acidergic (GABAergic) currents. Whether the net effect of active KCC2 is inhibitory via such Cl^−^ extrusion or excitatory due to the concomitant increase of K^+^ in the extracellular space is context‐dependent and difficult to predict. Consecutively, in rodent models, antiseizure‐ as well as seizure‐facilitating effects of KCC2 block have been reported. Here, we attempted to gain more insight into KCC2's role in the seizure propagation zone in human temporal neocortex.

**Methods:**

We induced network activity in postoperative acute neocortical brain slices from humans with temporal lobe epilepsy under low Mg^2+^ conditions, with and without elevated K^+^, and recorded it using microelectrode arrays. We analyzed ictal‐like events and interictal‐like discharges with a developed source‐separating approach. Finally, we complemented these network‐related studies by patch‐clamp recordings of individual pyramidal neurons under regular ionic conditions to assess the inherent functionality of KCC2 and alternative transmembrane Cl^−^ routes.

**Results:**

Modulation of KCC2 activity altered the induced network activity; KCC2 block reversibly led to substantially increased activity, preferentially in and propagating through supragranular layers. Correspondingly, enhancing KCC2 activity reduced network activity there. Almost all individual supragranular pyramidal neurons tested had functional KCC2, that is, certain Cl^−^ extrusion capacity that was limited when loaded with higher Cl^−^ and presented variable predominantly positive values of GABA_A_ receptor driving force. In addition, we found tonic inhibition that increases after prolonged KCC2 block and may either contribute to Cl^−^ load or support Cl^−^ extrusion in supragranular pyramidal neurons, depending on their intracellular Cl^−^ concentration.

**Significance:**

Our data show that KCC2 mitigates ictal‐like activity in the seizure propagation zone of human neocortex, thereby further promoting KCC2 as a therapeutic target.

## Graphical abstract

The K⁺/Cl^−^ cotransporter (KCC2) limits induced ictal‐like events and their spread in the supragranular layer of human neocortical slices from the seizure‐propagation zone of temporal lobe epilepsy patients. Cl⁻ extrusion is present in supragranular pyramidal neurons but limited under high somatic Cl⁻ load. KCC2 blockade enhances tonic inhibition (TI), constituting an alternative transmembrane Cl⁻ route.

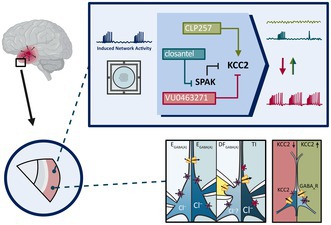

**Key points**

- KCC2 mitigates ictal‐like events and their propagation in supragranular layers of neocortical acute brain slices from the seizure propagation zone of TLE patients.
- Individual human pyramidal supragranular neurons have a limited capacity for Cl^−^ extrusion under somatic Cl^−^ load.
- The block of KCC2 leads to increased tonic inhibition, constituting an alternative transmembrane Cl^−^ route.

## 1 INTRODUCTION

The human supragranular neocortex has recently been shown to contain the network involved in the propagation and continuation of seizures in vivo.^1^ This renders nonfocal brain tissue from patients who have undergone anterior temporal lobe resections to treat drug‐resistant focal epilepsy worth studying, because full “ictal” events develop only when neuronal activity breaks away from the restricted focus.^2^ Although most of the time such escape is prevented by an inhibitory restraint,^3^ intensely active interneurons may elevate extracellular potassium ([K^+^]_*o*_) and intracellular chloride ([Cl^−^]_*i*_) in parallel. Either of these has been shown to initiate, maintain, and propagate epileptic seizures.

Transient activity‐dependent elevations of [K^+^]_*o*_ likely differ region‐ and layer‐specifically^4^ and constitute a classical focus of epilepsy research. [K^+^]_*o*_ depolarizes neuronal membranes, directly induces hyperexcitability, and results in the emergence of neuronal network resonance. Indirectly, augmented [K^+^]_*o*_ leads to a positive shift in γ‐aminobutyric acid type A (GABA_A_) reversal (*E*_GABA(A)_), thereby attenuating inhibition,^4^ and even tends to coordinate raised [Cl^−^]_*i*_ across local populations of neurons. [Cl^−^]_*i*_ directly determines the Cl^−^ reversal potential (*E*_*Cl*_ *−*), the Cl^−^ driving force (*DF*_*Cl*_ *−*), and consequently the efficacy of GABA_A_ receptor (GABA_A_R)‐mediated inhibition.^5^ Alterations in synaptic inhibition are an important component of epileptogenesis.^6^ GABA responses change polarity during prolonged GABA_A_R activation due to ongoing interneuronal firing.^7^, ^8^ The underlying collapse of the transmembrane electrochemical Cl^−^ gradient requires the depolarizing HCO_3_^−^‐mediated current component.^9^ The substantial HCO_3_^−^ permeability of GABA_A_R channels^10^ and the much less negative HCO_3_^−^ reversal (approximately −15 mV) result in an efflux of HCO_3_^−^ upon GABA_A_R activation.

Both [K^+^]_*o*_ and [Cl^−^]_*i*_ are coupled by the neuron‐specific K^+^/Cl^−^ cotransporter 2 (KCC2).^11^ KCC2 is regarded as the main neuronal Cl^−^ extruder and is driven by the K^+^ gradient.^12^ Many studies including those in mouse epilepsy models^13^, ^14^, ^15^ have revealed a proinhibitory action of functional KCC2 by Cl^−^ extrusion, which maintains hyperpolarizing inhibition. In line with this, enhancing KCC2 for instance by genetic modification of inhibitory KCC2 phosphorylation reduced seizure activity.^16^ On the other hand, KCC2 could act in a proexcitatory manner by facilitating the spread of epileptic activity due to [K^+^]_*o*_ increase, either directly because Cl^−^ extrusion via KCC2 inevitably leads to rising [K^+^]_*o*_ ^17^ or indirectly because reversed KCC2 action^18^ could further promote [Cl^−^]_*i*_ accumulation. Such a reversal could be due to epileptic activity and has been shown to lead to neuronal swelling and death.^19^ Consistently, pharmacological suppression of KCC2 abolishes 4‐aminopyridine‐induced ictal‐like activity, whereas KCC2 enhancement increases the duration of ictal‐like events (ILEs) in vitro in rodents and in silico.^20^, ^21^ To add more complexity, KCC2 not only regulates [K^+^]_*o*_ and [Cl^−^]_*i*_ but is also determined by them.^12^, ^18^, ^19^

Because theoretically KCC2 might act in a dual manner—by enhancing hyperpolarizing inhibition via Cl^−^ extrusion, thereby suppressing seizures, and by promoting neuronal excitability and epileptiform activity via K^+^ extrusion—and both antiseizure and seizure‐facilitating effects of KCC2 activity have been reported in rodents, the role of KCC2 in the human seizure propagation zone is hard to predict. Additionally, KCC2 function is context‐dependent, and network alterations in chronically epileptic human tissue may differ from those in animal models. Our in vitro study of functional effects of KCC2 modulation on the level of network activity and individual neurons supports protective KCC2 function^22^ also in the human seizure propagation zone.

## 2 MATERIALS AND METHODS

### 2.1 Human neocortical tissue

Neocortical tissue samples from the anterior middle temporal gyrus of 36 patients (13 female, 23 male) were included. Thirty‐one patients suffered from drug‐resistant temporal lobe epilepsy (TLE) without a tumor, and five patients with TLE had a tumor in the temporal lobe (Table 1). The study was approved by the local ethics committee (Berlin: EA2/111/14, Bielefeld: 2020‐517‐f‐S, Hamburg: 2023‐200 674‐BO‐bet). All patients gave written consent for the scientific use of their tissue. All procedures adhered to ethical requirements (Declaration of Helsinki).

**TABLE 1:** Patient characteristics.

Age, years	Sex	Epilepsy duration, years	Average frequency of seizures per month	Diagnosis	Antiseizure medication at time of resection
25–29	Female	5	3	Reactive gliosis	Lamotrigine, zonisamide
25–29	Female	16	24	Reactive gliosis	Oxcarbazepine
45–49	Male	12	5	Hippocampal sclerosis ILAE type 1	Lamotrigine
25–29	Female	9	3	Reactive gliosis	Lacosamide, levetiracetam
35–39	Female	38	30	Hippocampal sclerosis ILAE type 2	Clobazam, lamotrigine, pregabalin
20–24	Male	7	30	Reactive gliosis	Eslicarbazepine acetate, lamotrigine
25–29	Male	17	30	Hippocampal sclerosis ILAE type 2	Brivaracetam, lacosamide
20–24	Female	8	15	Reactive gliosis	Lamotrigine
20–24	Male	3	15	Reactive gliosis	Lacosamide, lamotrigine
30–34	Male	28		Reactive gliosis	Lamotrigine
20–24	Female	4	30	Hippocampal sclerosis ILAE type 1	Oxcarbazepine
45–49	Female	20	2	Hippocampal sclerosis ILAE type 1	Lacosamide
35–39	Male	16	30	Reactive gliosis	Clobazam, lacosamide
20–24	Female	6	20	Reactive gliosis	Clobazam, lamotrigine, levetiracetam
30–34	Male	12	3	Reactive gliosis	Lamotrigine, levetiracetam, perampanel
30–34	Male	14	2	Reactive gliosis	Lacosamide, oxcarbazepine
45–49	Male	8	6	Hippocampal sclerosis ILAE type 1	Gabapentin, lamotrigine
30–34	Male	8	5	Reactive gliosis	Clobazam, lamotrigine, oxcarbazepine, perampanel
50–54	Male	51	4	Hippocampal sclerosis ILAE type 1	Clobazam, lacosamide, lamotrigine
30–34	Male	6	2	Reactive gliosis	Clobazam, levetiracetam
45–49	Female	10	30	Hippocampal sclerosis ILAE type 1	Lacosamide
20–24	Male	3	30	Hippocampal sclerosis ILAE type 1	Clobazam, lacosamide
55–59	Male	8	12	Reactive gliosis	Clobazam, lamotrigine
15–19	Male	17	7	Hippocampal sclerosis ILAE type 1	Brivaracetam, lacosamide
60–64	Female	9	7	Hippocampal sclerosis ILAE type 3	Brivaracetam, perampanel
35–39	Male	11	1	Hippocampal sclerosis ILAE type 1	Lacosamide
70–74	Male	35	3	Hippocampal sclerosis ILAE type 1	Clobazam, Lacosamide, levetiracetam
20–24	Male	19	24	Hippocampal sclerosis ILAE type 1	Clobazam, levetiracetam, oxcarbazepine
40–44	Female	35	10	Hippocampal sclerosis ILAE type 1	Lamotrigine
50–54	Female	41	12	Hippocampal sclerosis ILAE type 1	Brivaracetam, cenobamate
50–54	Female	3	3	Reactive gliosis	Lacosamide
20–24	Male	1	12	Dysembryoplastic neuroepithelial tumor CNS WHO grade I	
20–24	Male	8	13	Dysembryoplastic neuroepithelial tumor CNS WHO grade I	Lacosamide
20–24	Male	4	16	Ganglioglioma, CNS WHO grade I	Clobazam, lamotrigine, levetiracetam
15–19	Female	4	5	Low‐grade neuroepithelial tumor	Brivaracetam, clobazam
30–34	Male	8	12	Dysembryoplastic neuroepithelial tumor CNS WHO grade I	Lacosamide

*Note*: Age is given as a 5‐year range to grant greater anonymity. The median seizure frequency was 12 per month (interquartile range = 18.5, *n* = 36), and 19.4% of patients experienced daily seizures before surgery.

Abbreviations: CNS, central nervous system; ILAE, International League Against Epilepsy; WHO, World Health Organization.

### 2.2 Slice preparation

Tissue was collected in the operating theater, handled as previously described,^23^ transported, and cut into 300‐μm slices in sucrose‐containing artificial cerebrospinal fluid (sACSF) at 2–4°C on a vibratome (Leica VT1200S). Slices recovered in sACSF for 30 min at 32°C were transferred to hydroxyethylpiperazine ethane sulfonic acid‐containing ACSF (hACSF) and kept at room temperature until recording. Extracellular solutions were carbonated (95% O_2_/5% CO_2_) and equilibrated at pH 7.4. See Table S1 for solutions and Table S2 for chemicals.

### 2.3 Microelectrode array recordings

The 60EcoMEA (Multi Channel Systems) consists of 59 electrodes and one reference electrode in an 8 × 8 layout grid, with an electrode diameter of 100 μm and an electrode spacing of 700 μm. Activity was recorded with the microelectrode array (MEA) 2100—120 system (bandwidth = 1–3.000 Hz, Multi Channel Systems Experimenter) and sampled at 10 kHz. Experiments were performed at 32°C. ILEs were induced by perfusion with modified ACSF lacking Mg^2+^ or containing .25 mmol·L^−1^ Mg^2+^ and 6 mmol·L^−1^ or 3 mmol·L^−1^ K^+^. Slices were perfused at 5–6 mL/min with the modified ACSF for a minimum of 22 min before recording (timelines are provided in Figures S1 and S2A,C). After 20 min of stable baseline recording, KCC2 modulators VU0463271 (10 μmol·L^−1^), closantel (10 μmol·L^−1^), or CLP257 (50 μmol·L^−1^) were bath applied.

**FIGURE 1:**
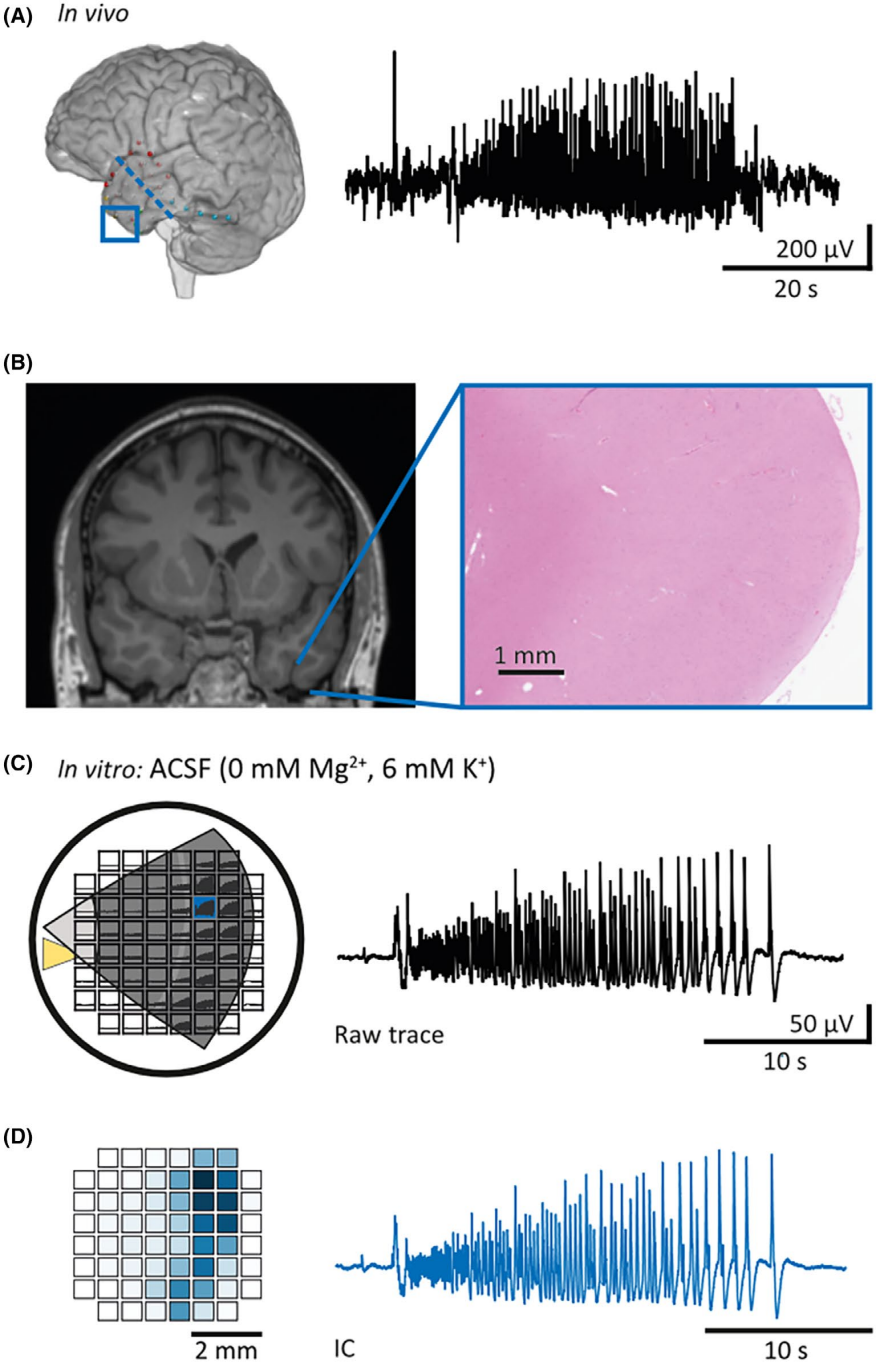
Phenomenological resemblance of in vivo ictal events and induced in vitro ictal‐like events (ILEs). (A) Magnetic resonance imaging (MRI)‐based three‐dimensional reconstruction and exemplary preoperative intradural electroencephalography recording of a spontaneous ictal event recorded in the framed area (seizure propagation zone) during a focal seizure. The blue dashed line represents the resection margin. (B) Preoperative frontal MRI T1 sequence and exemplary postoperative neocortical microscopy (hematoxylin and eosin stained) of the resected temporal pole showing reactive changes without further pathology in the neuropathological workup. (C) Left: Position of the neocortical slice from the medial temporal gyrus on the microelectrode grid. ILEs as induced by proictal artificial cerebrospinal fluid (ACSF; 0 mmol·L^−1^ Mg^2+^, 6 mmol·L^−1^ K^+^) occur and propagate in neocortical supragranular layers. Right: Raw voltage trace of one ILE, recorded by the marked (blue) electrode. The yellow triangle represents the reference electrode. When present, ILEs had a mean number of 8.9 per 20 min (95% credible interval [CrI] = 4.5–13.5), a mean spatial distribution across 21.2 electrodes (95% CrI = 14.6–27.9), and a mean duration of 22.6 s (95% CrI = 10.1–34.8). The mean frequency of discharges within ILEs was 3.1 s^−1^ (95% CrI = 2.4–3.7). (D) Left: Heat map of independent component analysis (ICA) weights (ratio of the raw signal attributed to the independent component [IC] of one source) indicating the electrodes involved in this ILE. Right: The IC represents the source of ictal‐like network activity of this recording. IC traces capturing the entire recorded network activity, as opposed to individual electrodes chosen by an analyst, were used in all microelectrode array analyses in this study. Note that the IC trace resembles the raw voltage trace of the electrodes with the highest ICA weights. The data presented are from one patient, highlighting the phenomenological resemblance of induced ILE evolution in vitro and spontaneous ictal activity in vivo.

A pipeline specifically developed for this analysis (inspired by established electroencephalographic [EEG] data analysis methods^24^) enabled the identification of groups of electrodes that exhibit coherent activity patterns during ILEs (Figure 1D). This approach reduces dimensionality, identifies individual sources, and subsequently allows analysis of the collective activity of each source, as opposed to raw data from individual electrodes (Figure S1A). The pipeline consists of data preprocessing, followed by principal component analysis and independent component analysis (ICA). An adaptation of the maxed interval method,^25^ commonly used for burst detection, is used for ILE detection. The pipeline identifies electrodes that are active during activity (spatial distribution), calculates the mean duration of ILEs, and determines the frequency of discharges within ILEs for each source. See Supporting Information methods for further information.

### 2.4 Patch‐clamp recordings

Slices were placed in a submerged‐type recording chamber constantly perfused with ACSF (KCl 2.5 mmol·L^−1^, MgCl_2_ 1.3 mmol·L^−1^, 32°C) at 2–3 mL/min. Supragranular pyramidal neurons were recorded using an EPC10‐USB amplifier with the respective software (Patchmaster, HEKA). Data were filtered at 2.9 kHz and sampled at 10 kHz. Pipettes were pulled from lead (PG10165, Precision Instruments, York, UK) or borosilicate glass (GB150‐10P, Science Products) using a Micropipette Puller (P‐97, Sutter Instruments) to 2–6 MΩ when filled with pipette solution (Table S2).

Neurons were somatically recorded in whole‐cell configuration with pipette solutions containing either 6, 19, or 41 mmol·L^−1^ Cl^−^. Cell capacitance was estimated from the integral and series resistance (*R*_S_) from the peak current of the slow capacitive transient upon a −10‐mV rectangular voltage pulse. To assess KCC2 extrusion capacity, neurons were voltage‐clamped (−80 to −40 mV, increments of 5 or 10 mV), and GABA_A_ responses were evoked at dendrite and soma by pressure ejection of ACSF with GABA (10 μmol·L^−1^). Amplitude of GABA_A_ currents (*I*_GABA(A)_) was plotted against the comand voltage (*V*_*p*_), and *E*_GABA(A)_ was determined at *I*_GABA(A)_ = 0 at the soma (*E*_GABA(A) soma_) and dendrite (*E*_GABA(A) dendrite_), respectively. The positive correlation between *E*_GABA(A)_ at soma and dendrite (repeated measures correlation: *r* = .82, *df* = 32, 95% confidence interval [CI] = .67–.91, number of experiments [*n*] = 44, number of patients [*N*] = 11; Figure 4E) indicates sufficient distribution of the loaded Cl^−^. The Goldman–Hodgkin–Katz equation was used to estimate *E*_*Cl*_^*−*^ at soma and dendrite, respectively (with [HCO_3_]_*i*_^−^ = 16.38 mmol·L^−1^, calculated with the Henderson–Hasselbalch equation at an pH_i_ of 7.2 and extracellular [HCO_3_]_*o*_^−^ of 26 mmol·L^−1^ at pH_o_ of 7.4, according to Kaila^7^ and Farrant and Kaila,^26^ and relative permeability of HCO_3_^−^ vs. Cl^−^ of .3). Likewise, theoretical *E*_GABA(A) soma_ values were calculated from the [Cl^−^]_*p*_ (i.e., 19 or 41 mmol·L^−1^).

Tonic GABA_A_R currents were recorded in neurons clamped at −60 mV. D(−)‐2‐Amino‐5‐phosphonopentanoic acid (25 μmol·L^−1^) and 6‐cyano‐7‐nitroquinoxaline‐2,3‐dione (20 μmol·L^−1^) were added to block excitatory inputs, and (−)‐bicuculline methiodide (BMI; 10 μmol·L^−1^) was added to block phasic and tonic GABA_A_ receptors. Tonic GABA_A_R currents were calculated as current differences before and after the application of BMI. Recordings in which *R*_*s*_ change exceeded 20% were excluded. Liquid junction potential was calculated (6 mmol·L^−1^ [Cl^−^]_*p*_: 14.7 mV, 19 mmol·L^−1^ [Cl^−^]_*p*_: 13.4 mV, and 41 mmol·L^−1^ [Cl^−^]_*p*_: 11.3 mV, tonic inhibition trials: 2.7 mV; JPCalc by Peter Barry, University of New South Wales) and corrected for offline. Data were analyzed using FitMaster v2x90.4 (HEKA), AxographX1.8.0 (Axograph Scientific), and OriginPro 2022 (OriginLab Corporation).

### 2.5 Statistical analysis

MEA recordings, KCC2 extrusion capacity, and tonic inhibition were analyzed pairwise. Values are expressed as median with interquartile range. Because the data of this study include multiple measurements from the same individual, independence (a prerequisite for classical hypothesis tests, e.g., *t*‐tests) cannot be assumed. Therefore, Bayesian linear mixed‐effects models with random intercepts were used to address the hierarchical data structure and accommodate both within‐subject correlations and between‐subject variability.^27^ Unless stated otherwise, effect sizes represent mean differences derived from these models and are estimated as means of the posterior distribution along with 95% credible intervalls (CrI). To account for baseline influences in the MEA experiments, estimates were adjusted for the average baseline in KCC2 enhancement experiments and for either no activity or baseline activity in KCC2 block experiments. An additional model with an added fixed effect was used to analyze the influence of preoperative seizure frequency on the outcome parameters. Adjusted intraclass correlation coefficients were calculated. The association between *E*_GABA(A) soma_ and *E*_GABA(A) dendrite_, as well as cell depth and DF_GABA(A)_, was assessed using repeated‐measures correlation coefficients. Probability values are not reported, as they are incompatible with Bayesian concepts and have widely recognized limitations.^28^, ^29^ The number of experiments (i.e., neurons for patch‐clamp recordings and slices for MEA experiments) is indicated as “*n*”, the number of patients as “*N*”. See Supporting Information methods for further information on statistical methods.

## 3 RESULTS

### 3.1 Pharmacological KCC2 modulation opposingly alters human neocortical network activity

We investigated the effects of KCC2 modulation on induced network activity with MEAs in 91 acute neocortical brain slices from 18 patients (Figures 1 and S2). To induce ILEs, we modified our ACSF by omitting Mg^2+^ and slightly elevating K^+^ (0 mmol·L^−1^ Mg^2+^/6 mmol·L^−1^ K^+^) in most experiments (*n* = 71, *N* = 13). Lowering Mg^2+^ has been shown to be particularly helpful in providing insight into the spread of seizure activity.^3^, ^30^ Notably, we did not block K^+^ channels, to avoid uncontrolled [K^+^]_*o*_ accumulation. As expected for tissue from patients with a history of epilepsy, this commonly used method led to ILEs in 41 of 72 slices (in 11/13 patients). The in vitro recorded ILEs resembled our (Figure 1A) and previously described patterns of ictal activity identified by intracranial EEG recordings.^31^ Notably, features of epileptiform activity (ictal‐ and interictal‐like) were comparable to previous recordings of epileptic human temporal lobe.^32^, ^33^ In particular, the ILE structure—preceded by large‐amplitude preictal discharges—mirrored the one described in human tissues such as dysplastic cortex,^34^ subiculum,^33^, ^35^ or peritumoral cortex.^36^ Specifically, 56% of ILEs presented as low‐voltage fast onset patterns, whereas 44% were consistent with the hypersynchronous pattern. We objectively and comprehensively analyzed our multidimensional MEA data with the ICA pipeline (Figures 1D, S1A, and Supporting Information methods). As previously deduced from in vivo laminar electrode recordings,^1^ ILEs occurred in supragranular layers (41/41). In three slices, we additionally recorded ILEs in lower layers.

**FIGURE 2:**
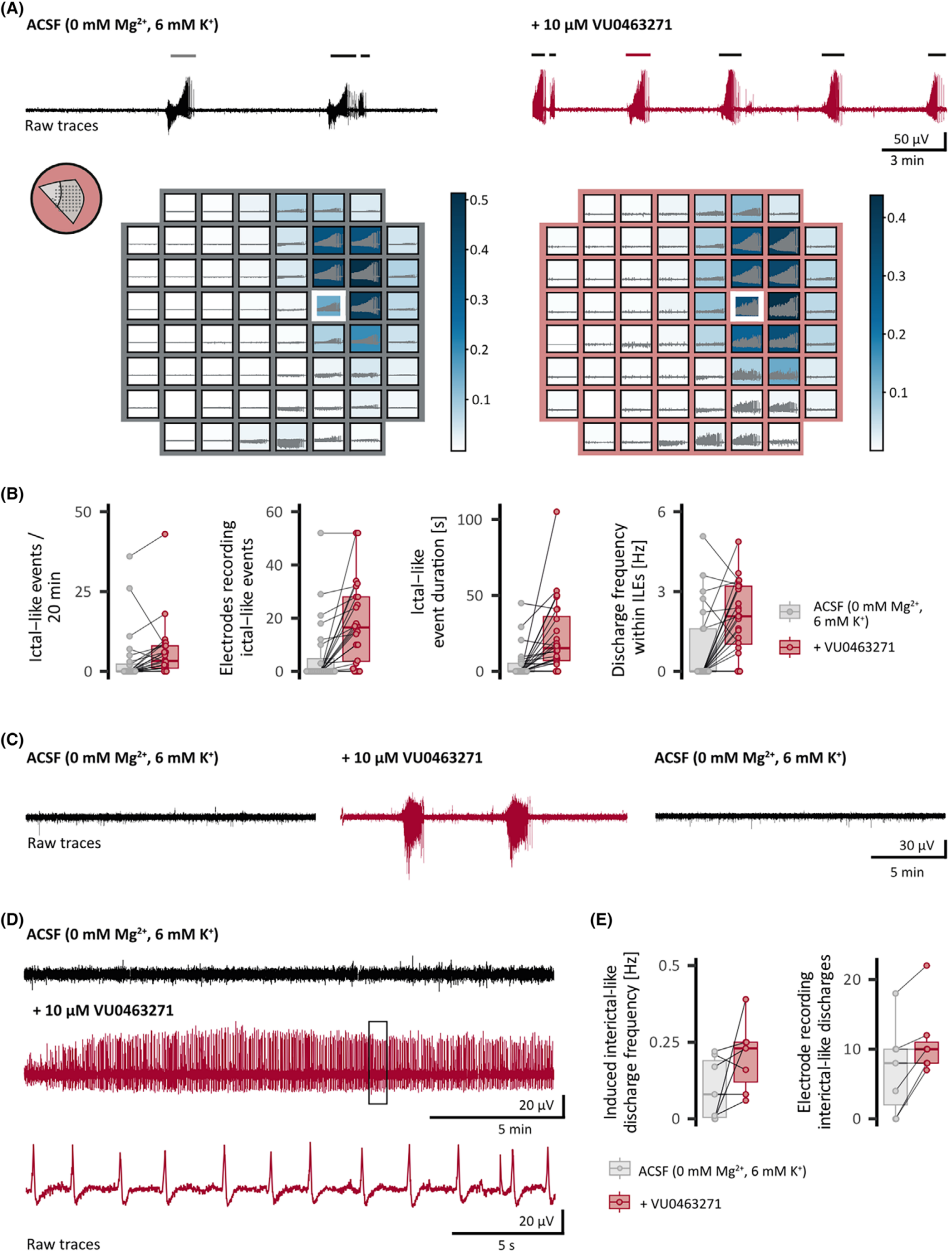
Block of K^+^/Cl^−^ cotransporter 2 (KCC2) enhanced ictal‐like events (ILEs) and interictal‐like discharges (IILDs) in acute neocortical slices from patients with temporal lobe epilepsy. (A) Raw voltage traces and spatial heat maps of one neocortical brain slice before (left) and under (right) application of the specific KCC2 blocker VU0463271 (10 μmol·L^−1^). Top: Raw voltage trace before (black) and under KCC2 block (red) recorded by the white‐framed electrode. The bars on top of the traces indicate ILEs. Bottom: Heat maps of independent component analysis weights (ratio of the raw signal attributed to the independent component of one of the two sources of ILEs in this slice) and raw voltage traces of corresponding electrodes for the ILE highlighted above (gray/red). Discharges at the lower right corner of the electrode grid represent the second source of activity. Inset: Slice position on electrode grid. (B) Population data. KCC2 block increased the number of ILEs during the 20‐min analysis window (descriptive statistics: from .0, interquartile range [IQR] = 2.2 to 3.2, IQR = 7.0, number of experiments [*n*] = 24, number of patients [*N*] = 12), the number of electrodes recording ILEs (descriptive statistics: from .0, IQR = 4.8 to 16.6, IQR = 24.4, *n* = 24, *N* = 12), the duration of ILEs (descriptive statistics: from .0 s, IQR = 5.3 to 15.2 s, IQR = 28.9, *n* = 24, *N* = 12), and the frequency of discharges within ILEs (descriptive statistics: from .0 s^−1^, IQR = 1.6 to 2.1 s^−1^, IQR = 2.0, *n* = 24, *N* = 12). In 13 slices, de novo ILEs developed upon KCC2 block. (C) Effects of KCC2 block on ILEs were reversible. Top: Raw voltage traces before (black), during (red), and after application of VU0463271 (black). Washout decreased the number of ILEs/20 min in all experiments (descriptive statistics: from 9.0, IQR = 4.0 to .0, IQR = 3.0, *n* = 5, *N* = 2; in 60% it abolished ILEs) and the spatial extent of ILEs (descriptive statistics: from 18.0 electrodes, IQR = 7.0 to .0 electrodes, IQR = 17.0, *n* = 5, *N* = 2). (D) KCC2 block increased IILDs in slices or independent foci that did not exhibit ILEs. Example traces before (top, black) and upon (middle, red) application of VU0463271 and magnification of IILDs at the marked timeframe (bottom, red) are shown. Population data depict changes in the frequency of IILDs (descriptive statistics: from .08 s^−1^, IQR = .18 to .23 s^−1^, IQR = .13, *n* = 7, *N* = 4) and number of electrodes recording IILDs (descriptive statistics: from 8.0, IQR = 8.0 to 10.0, IQR = 3.0, *n* = 7, *N* = 4). Estimated effect sizes are displayed along with the effects of KCC2 enhancement in Figure 3A. ACSF, artificial cerebrospinal fluid.

**FIGURE 3:**
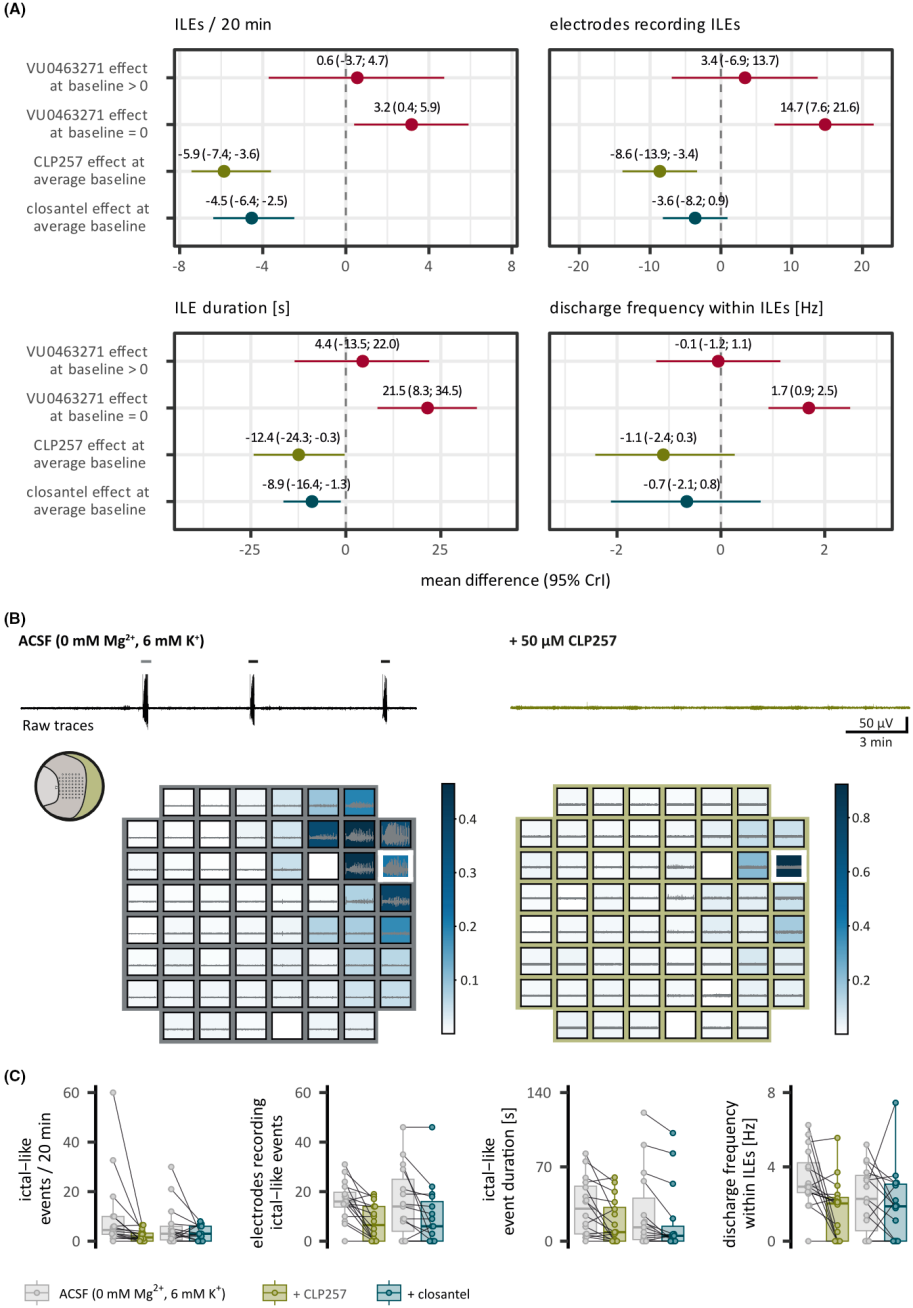
Opposing effects of K^+^/Cl^−^ cotransporter 2 (KCC2) manipulations. KCC2 enhancers diminished ictal‐like events (ILEs) in acute neocortical slices from patients with temporal lobe epilepsy. (A) Forest plots of mean difference with 95% credible intervals (CrIs) before and under KCC2 block or enhancement, derived from Bayesian mixed effects models. We adjusted for baseline activity, as effect sizes depended on baseline activity (Figure S3D). KCC2 blocker (VU0463271, red) was applied to slices with (>0) and without (=0) baseline activity, and results are displayed separately. The effects of direct KCC2 enhancer (CLP257, green) and indirect KCC2 enhancer (closantel, teal) are displayed for the mean baseline value. KCC2 block increased the number of ILEs, electrodes recording ILEs, duration of ILEs, and discharge frequency of ILEs. The effect was diminished or less pronounced when ILEs were present at baseline. Conversely, the KCC2 enhancers CLP257 and closantel reduced the outcome parameters. However, the decrease in the spatial distribution of ILEs under closantel was less robust, and closantel did not relevantly change the discharge frequency within ILEs. (B) Top: Exemplary raw voltage traces and spatial heat maps of one neocortical brain slice before (left) and under (right) application of the KCC2 enhancer CLP257. Traces (black/green) were recorded from the white‐framed electrode. The bars on top of the traces indicate ILEs. Bottom: Heat maps of independent component analysis weights (ratio of the raw signal attributed to the independent component of the source of ILEs in this slice) and raw voltage traces of corresponding electrodes for the ILE highlighted above the trace (gray/green). Inset: Slice position on the electrode grid. (C) Population data of outcome parameters before and under KCC2 enhancement. CLP257 (green) decreased the number of ILEs/20 min (descriptive statistics: from 4.5, interquartile range [IQR] = 7.2 to 1.5, IQR = 3.2, number of experiments [*n*] = 16, number of patients [*N*] = 4), the number of electrodes recording ILEs (descriptive statistics: from 16.0, IQR = 6.0 to 6.5, IQR = 14.0, *n* = 16, *N* = 4), the frequency of discharges within ILEs (descriptive statistics: from 2.9 s^−1^, IQR = 1.6 to 2.0 s^−1^, IQR = 2.4, *n* = 16, *N* = 4), and the duration of ILEs (descriptive statistics: from 30.6 s, IQR = 45.0 to 8.5 s, IQR = 31.8, *n* = 16, *N* = 4). Closantel (teal) decreased the number of electrodes recording ILEs (descriptive statistics: from 14.0, IQR = 21.0 to 6.0, IQR = 16.0, *n* = 15, *N* = 5) and the duration of ILEs (descriptive statistics: from 13.0 s, IQR = 39.0 to 5.8 s, IQR = 14.0, *n* = 15, *N* = 5), and to a less pronounced extent, the number of ILE (descriptive statistics: from 3.0, IQR = 5.0 to 3.0, IQR = 6.0, *n* = 15, *N* = 5) and the frequency of discharges within the ILEs (descriptive statistics: from 2.3 s^−1^, IQR = 3.0 to 1.9 s^−1^, IQR = 3.1, *n* = 15, *N* = 5). ACSF, artificial cerebrospinal fluid.

Assessing the role of KCC2 in maintaining ionic balance in the human neocortical circuit, that is, whether the antiseizure [Cl^−^]_*i*_ reduction outweighs the inevitable [K^+^]_*o*_ increase, we first applied the selective KCC2 antagonist VU0463271 (10 μmol·L^−1^)^13^, ^37^ to unravel the consequences of KCC2 block on the induced network activity. The resulting KCC2 block increased network activity, pointing to relative predominance of [Cl^−^]_*i*_ reduction over [K^+^]_*o*_ accumulation. It relevantly increased the number (∆ = 2.4/20 min, 95% CrI = .1–4.8), spatial distribution (∆ = 11.5 electrodes, 95% CrI = 5.0–18.0), and duration (∆ = 19.3 s, 95% CrI = 11.6–29.9) of ILEs as well as the frequency of discharges within ILEs (∆ = 1.2 s^−1^, 95% CrI = .5–1.9; Figure 2A,B; *n* = 24, *N* = 12). Removing VU0463271 confirmed the causative nature of the KCC2 block; it reduced the frequency and spatial distribution of ILEs (Figure 2C; *n* = 5, *N* = 2; in 60%, it abolished ILEs). Strikingly, KCC2 block triggered de novo ILEs in supragranular layers (Figure 2C). In addition to ILEs, there were synchronous interictal‐like discharges (IILDs) that did not meet our criteria for ILEs (Supporting Information methods). We recorded such IILDs between ILEs, independently in another independent component or exclusively. As during postictal depression in vivo, IILDs decreased immediately after ILEs. Accordingly, we only analyzed IILDs that occurred independently of ILEs (Figure 2D; *n* = 7, *N* = 4). In line with the effect on ILEs, blocking KCC2 increased the spatial distribution of IILDs (∆ = 4.55 electrodes, 95% CrI = .9–8.2) and tended to increase the average frequency of IILDs (∆ = .12 Hz, 95% CrI = −.2 to .5), although this is to be interpreted with caution due to the small sample size of this subanalysis. KCC2 block had a less robust or no effect on ILE parameters when baseline ILEs were present in modified ACSF (Figure 3A). This dependence of the KCC2 block on baseline ILEs could suggest a reciprocal connection between preexisting KCC2 function and ILEs.

Notably, the effect of the KCC2 block on ILEs did not depend on the composition of our proictal ACSF. Neither elevating Mg^2+^ to .25 mmol·L^−1^ (because 0 mmol·L^−1^ [Mg^2+^]_*o*_ ACSF may lead to a massive influx of Ca^2+^; Figure S2A,B) nor lowering K^+^ to 3 mmol·L^−1^ in 0 Mg^2+^ ACSF (because 6 mmol·L^−1^ [K^+^]_*o*_ may attenuate KCC2; Figure S2C,D) qualitatively changed the augmenting effect of VU0463271 on ILE number and spatial distribution.

If KCC2 activity is critically involved in synchronous activity in the human neocortex, KCC2 amplification should reduce ILEs. We tested the effect of two different potential KCC2 enhancers because there was no selective pharmacological activator of KCC2 at the beginning of our study.^38^, ^39^ The level of ILE activity in control recordings had a relevant effect on the outcome; the more activity under control conditions, the greater the effect of the KCC2 enhancers (Figure S3D). This supports the idea of a link between KCC2 function and ILEs, as neurons with lower KCC2 function may have more potential for its enhancement. CLP257, which enhances KCC2 but also extrasynaptic GABA_A_R,^38^, ^39^, ^40^ decreased the number of ILEs, spatial distribution, duration, and less robustly the discharge frequency within ILEs (Figure 3A–C). Likewise, closantel, which indirectly enhances KCC2 by inhibiting the with‐no‐lysine (WNK) kinase‐STE20/SPS1‐related proline/alanine‐rich kinase (SPAK) pathway and thereby disinhibiting KCC2,^41^ relevantly reduced the number of ILEs and ILE duration and less robustly the spatial distribution of ILEs. Closantel did not relevantly alter the discharge frequency within ILEs (Figures 3A,C and S3A). Additionally, closantel tended to reduce IILDs; however, we did not perform inferential statistics due to the small sample size (*n* = 5, *N* = 3; Figure S3B,C). Note that we cannot exclude a partial contribution of NKCC1 to the closantel effect as has been shown for other WNK kinase inhibitors.^22^ (For further details on closantel actions on NKCC1, see Supporting Information methods.) However, the qualitatively similar outcome upon both substances is caused by the linking feature (KCC2 enhancement) rather than by the diverse other actions.

Both blocker and enhancer effects not only support current intensive efforts to develop KCC2‐directed therapies^42^ but also point to the existence/availability of Cl^−^ extrusion by modifiable KCC2 in neocortical tissue of patients suffering from TLE.

### 3.2 Capacity of Cl^−^ extrusion is limited as shown by artificially increased [Cl^−^]_*i*_ in individual human supragranular neocortical pyramidal neurons

**FIGURE 4:**
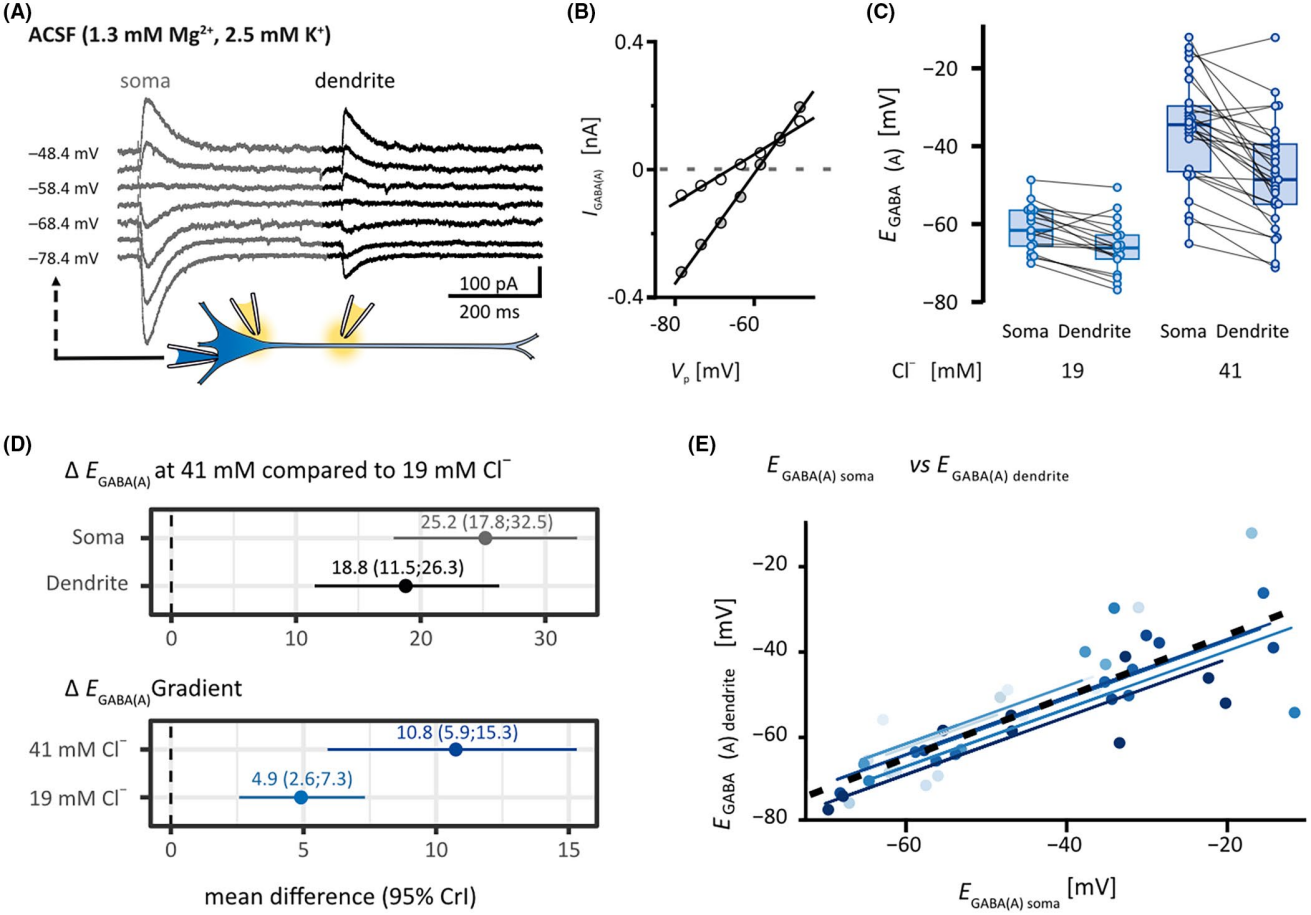
Cl^−^ extrusion capacity did not restore γ‐aminobutyric acid type A (GABA_A_) receptor reversal potential (*E*_GABA(A)_) when somatic Cl^−^ loading elevated [Cl^−^]_*i*_ in supragranular pyramidal neurons of patients suffering from temporal lobe epilepsy. (A) Top: GABA_A_ receptor currents at −48.4 to −78.4 mV at 19 mmol·L^−1^ [Cl^−^]_*p*_ evoked by GABA (10 μmol·L^−1^) pressure ejection at the soma (gray) and at the dendrite (black). Bottom: Illustration of a somatic whole‐cell recorded neuron with pressure ejection sites. (B) Corresponding plot of currents at different membrane potentials (*V*_*p*_) evoked at the soma (gray) and the dendrite (black). *E*_GABA(A)_ = *V*_*p*_ when *I* = 0 (dotted gray line). (C) Population data of *E*_GABA(A)_ at soma and dendrite at nominally 19 mmol·L^−1^ [Cl^−^]_*p*_ (number of experiments [*n*] = 17, number of patients [*N*] = 8) or 41 mmol·L^−1^ [Cl^−^]_*p*_ (*n* = 27, *N* = 9). (D) Forest plots of mean difference and 95% credible intervals (CrIs), derived from Bayesian mixed effects models. At 41 mmol·L^−1^ [Cl^−^]_*p*_ somatic and dendritic *E*_GABA(A)_ and *E*_Cl_‐ were more depolarized compared to 19 mmol·L^−1^ [Cl^−^]_*p*_, yielding median *E*_GABA(A) soma_ and *E*_Cl_‐_soma_ of −61.6 mV, interquartile range [IQR] = 9.2 and −73.4 mV, IQR = 15.3 at 19 mmol·L^−1^ [Cl^−^]_*p*_, with −34.5 mV, IQR = 16.8 and −36.0 mV, IQR = 20.2 at 41 mmol·L^−1^ [Cl^−^]_*p*_. Additionally, median *E*_GABA(A) dendrite_ and *E*_Cl_‐_dendrite_ were −66.1 mV, IQR = 6.2 and −81.4 mV, IQR = 11.7 at 19 mmol·L^−1^ [Cl^−^]_*p*_ with −48.6 mV, IQR = 15.5 and −54.3 mV, IQR = 20.3 at 41 mmol·L^−1^ [Cl^−^]_*p*_, respectively (descriptive statistics); *E*_GABA(A)_ and *E*_Cl_‐ were more depolarized at soma than at dendrite, yielding median ∆ *E*_GABA(A)_ and ∆ *E*_Cl_− (∆*E*_Cl_‐ = dendritic *E*_Cl_‐ – somatic *E*_Cl_‐) at 19 mmol·L^−1^ [Cl^−^]_*p*_ of 5.5 mV (IQR = 4.3) and 10.6 mV (IQR = 10.6), with ∆ *E*_GABA(A)_ and ∆ *E*_Cl_− at 41 mmol·L^−1^ [Cl^−^]_*p*_ of 9.7 mV (IQR = 9.4) and 12.4 mV (IQR = 13.1), respectively (descriptive statistics). The *E*_GABA(A)_ gradient approximates to a change of [Cl^−^]_*i*_ concentration of 2.2 mmol·L^−1^, IQR = 1.2 (at 19 mmol·L^−1^ [Cl^−^]_*p*_) and 8.0 mmol·L^−1^, IQR = 13.3 (at 41 mmol·L^−1^ [Cl^−^]_*p*_). The percentage of variance explained by the variance between patients is small in both groups (19 mmol·L^−1^: 7.7%, 41 mmol·L^−1^: 7.6%, intraclass correlation coefficients from mixed effect models), suggesting that most of the variability is at a cellular rather than interindividual level. (E) Scatter plot depicting the positive correlation between *E*_GABA(A)_ values at soma and dendrite, indicating sufficient cytoplasmic diffusion of Cl^−^. Repeated measures correlation: *r* = .82, *df* = 32, 95% confidence interval [CI] = .67–.91, *n* = 44, *N* = 11). A different color represents each patient. Parallel lines are fitted for each patient, with the dashed black line representing the overall regression. Distance between the somatic and dendritic ejection site, series resistance (*R*_*s*_), and cell capacity (C) were comparable at both 19 and 41 mmol·L^−1^ [Cl^−^]_*p*_ (descriptive statistics: distance ejection sites: 65.0 μm, IQR = 25.0 [19 mmol·L^−1^ [Cl^−^]_*p*_] vs. 65.5 μm, IQR = 14.0 [41 mmol·L^−1^ [Cl^−^]_*p*_]; *R*_*s*_: 9.0 MΩ, IQR = 4.1 [19 mmol·L^−1^ [Cl^−^]_*p*_] vs. 7.9 MΩ, IQR = 3.3 [41 mmol·L^−1^ [Cl^−^]_*p*_]; C: 177.7 pF, IQR = 99.5 [19 mmol·L^−1^ [Cl^−^]_*p*_] vs. 168.3 pF, IQR = 109.6 [41 mmol·L^−1^ [Cl^−^]_*p*_]). ACSF, artificial cerebrospinal fluid.

Our MEA recordings revealed a dampening role for KCC2 in the seizure propagation zone on the network level. We next wanted to gain insight into KCC2 function on the single neuron level and performed cell‐attached recordings of single GABA_A_R channel‐mediated currents in visually identified supragranular pyramidal neurons (Figure S5). However, because Cl^−^ extrusion capacity cannot be estimated when measuring *E*_GABA_ in resting neurons, we adopted the approved method optimized to detect KCC2 transport efficiency.^11^, ^43^ As this assay is based on a defined [Cl^−^]_*i*_ load via a whole‐cell pipette, we artificially loaded the soma of supragranular neocortical pyramidal neurons from patients suffering from TLE with either 19 mmol·L^−1^ or 41 mmol·L^−1^ Cl^−^ in the pipette solution ([Cl^−^]_*p*_). After equilibration, we estimated somatic and dendritic *E*_GABA(A)_ values by sequential local pressure‐ejection of GABA at soma and dendrite. The observed somatodendritic *E*_GABA(A)_ gradient (∆*E*_GABA(A)_ = dendritic *E*_GABA(A)_ − somatic *E*_GABA(A)_) was generated by the activity of KCC2 and provides an estimate of the efficacy of extrusion.^11^, ^43^ Because HCO_3_^−^ has a substantial effect on *E*_GABA(A)_, especially at low levels of [Cl^−^]_*i*_, we provide the corresponding *E*_Cl_− for each *E*_GABA(A)_ value. Acute somatic load did not alter intrinsic neuronal properties of the recorded pyramidal neurons (Figure S4). Nevertheless, dendritic KCC2, if present and functional, should hyperpolarize *E*_GABA(A)_ at the dendrite compared to the soma, where [Cl^−^]_*i*_ was nominally determined by [Cl^−^]_*p*_.^11^ In both conditions (19 or 41 mmol·L^−1^ [Cl^−^]_*p*_), *E*_GABA(A)_ and *E*_Cl_− were more depolarized at the soma than at the dendrite (Figure 4C,D). Neurons loaded with 41 mmol·L^−1^ [Cl^−^]_*p*_ expectedly had a depolarized *E*_GABA(A) soma_ and *E*_Cl_− compared to those loaded with 19 mmol·L^−1^ (Figure 4D). Enhanced Cl^−^ load increased the difference between the *E*_GABA(A)_ and *E*_Cl_− gradient at 41 and 19 mmol·L^−1^ (∆_41–19 mM *E*GABA(A) gradient_ = 6.4 mV, 95% CrI = .8–12.0 and ∆_41–19 mM *ECl*_ *−*_gradient_ = 5.1 mV, 95% CrI = −2.0 to 12.35; for 19 mmol·L^−1^: *n* = 17, *N* = 8, for 41 mmol·L^−1^: *n* = 27, *N* = 9), yet dendritic *E*_GABA(A)_ and *E*_Cl_− remained more depolarized than observed with a lower Cl^−^ load (Figure 4D). Functional Cl^−^ extrusion in almost all neurons tested suggests the presence of functional KCC2 in the seizure propagation zone of TLE patients. In other words, although our group size is relatively small, we did not detect a major proportion of KCC2‐negative neurons as found in the subiculum^35^ or in the peritumoral zone.^36^ Together with the hint that the KCC2 extrusion capacity collapses with rising [Cl^−^]_*i*_, this provides a basis of KCC2 enhancement therapy also for the prevention of seizure spread.

### 3.3 Tonic inhibition in individual human TLE supragranular neocortical pyramidal neurons increases upon prolonged KCC2 block

**FIGURE 5:**
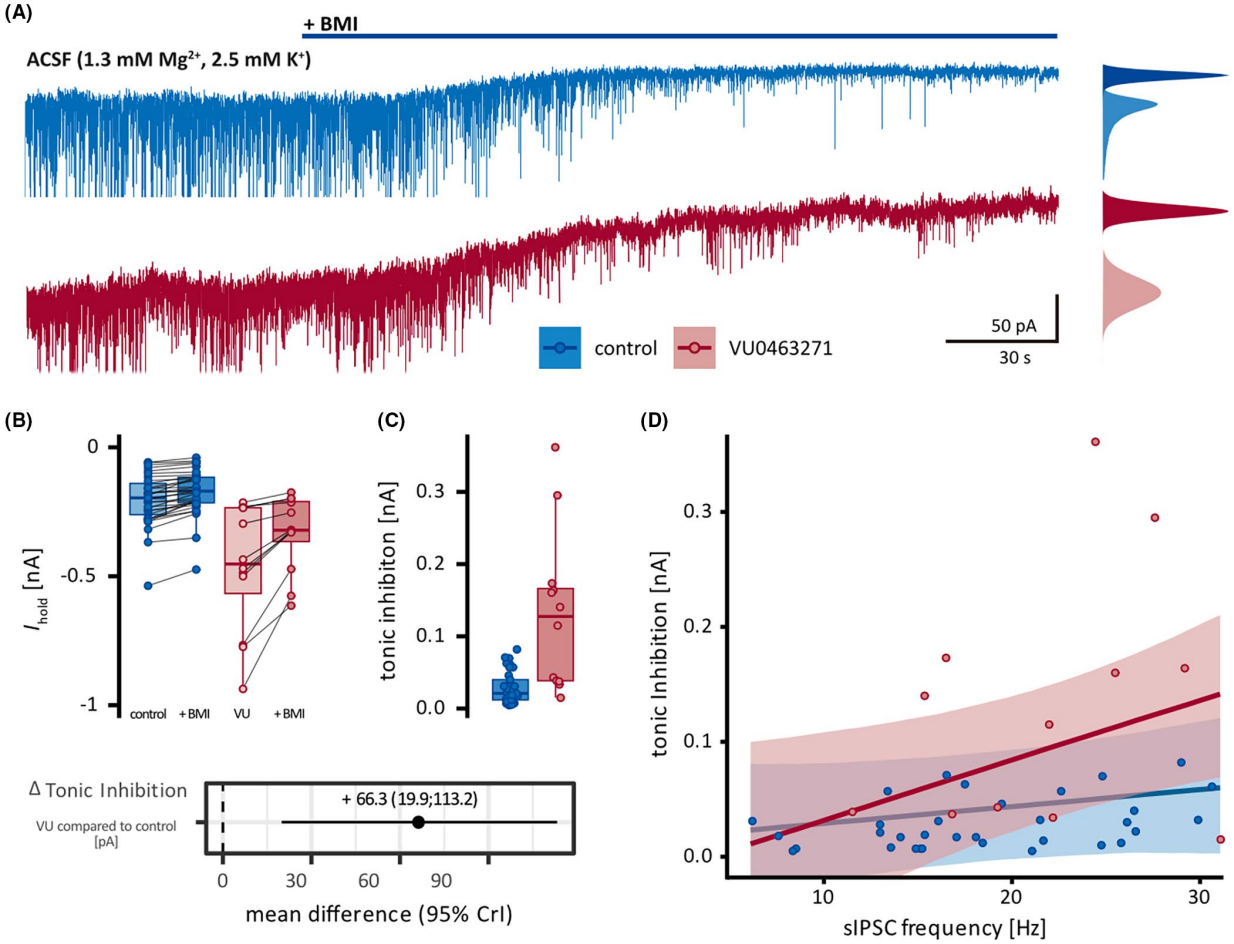
Tonic inhibition in supragranular neocortical pyramidal neurons of TLE patients increases after prolonged K^+^/Cl^−^ cotransporter 2 (KCC2) block. (A) Left: Examples of the effect of γ‐aminobutyric acid type A receptor blocker bicuculline (BMI; 10 μmol·L^−1^, black bar) on the somatically recorded holding current from a neuron filled with a CsCl‐based solution and voltage clamped at −73 mV in the presence of glutamate receptor blockers 6‐cyano‐7‐nitroquinoxaline‐2,3‐dione (20 μmol·L^−1^) and D(−)‐2‐amino‐5‐phosphonopentanoic acid (25 μmol·L^−1^) under control conditions (upper trace, blue) or after 2 h of exposure to VU0463271 (lower trace, red). Large phasic events were cut for clarity. Right: All‐point current histogram. Peaks were taken to estimate the holding current, and the peak difference was taken as the measure of tonic inhibition. (B, C) Upper panel: Population data of BMI‐mediated changes in holding current (B) *I*_hold_, before (light blue or light red, respectively) and under BMI (blue or red, respectively). (C) BMI uncovers an estimated mean tonic inhibition of 33.1 pA (95% credible interval [CrI] = 2.7–64.5; descriptive statistics: from −196.0 pA, interquartile range [IQR] = 121.0 to −170.0, IQR = 99.0, ∆ = 21.0 pA, IQR = 28.0, number of experiments [*n*] = 33, number of patients [*N*] = 8) under control condition (blue). Two hours of exposure to VU0463271 (10 μmol·L^−1^, red, left) tripled the BMI‐sensitive holding current component to 99.4 pA (95% CrI = 50.4–145.1; descriptive statistics: from −452.5 pA, IQR = 332.2 to −321.5 pA, IQR = 155.2, ∆ = 127.5 pA, IQR = 127.8, *n* = 12, *N* = 3). This corresponds to an increase in tonic inhibition of 66.3 pA (95% CrI = 19.9–113.2) when slices were pre‐exposed to VU0463271 (lower panel). The tonic inhibition density (tonic inhibition/cell capacity) was .11 pA/pF (95% CrI = .05–.18) for control and .23 pA/pF (95% CrI = .14–.31) after VU0463271 exposure. Series resistance (*R*_*s*_) and cell capacity (C) were comparable before and during BMI application (control: *R*_*s*_: 8.1 MΩ, IQR = 2.9 [before] vs. 8.4 MΩ, IQR = 3.5 [during BMI application]; C: 272.0 pF, IQR = 155.0 [before] vs. 246.5 pF, IQR = 164 [during BMI application]; VU0463271: *R*_*s*_: 9.8 MΩ, IQR = 4.3 [before] vs. 10.4 MΩ, IQR = 4.0 [during BMI application]; C: 442.0 pF, IQR = 155.5 [before] vs. 423.5 pF, IQR = 111.5 [during BMI application]). (D) Conditional effect plot. The main line represents the estimated effect of sIPSC frequency on tonic inhibition; the shaded area represents the uncertainty (95% CrI) of the effect in control condition (blue) or after VU0463271 exposure (red). A larger sIPSC frequency was accompanied by a larger tonic inhibition without and with KCC2 block (control: +1.38 pA per 1‐s^−1^ increase of sIPSC frequency, 95% CrI = −1.3 to 4.1; VU0463271: + 5.27 pA, 95% CrI = 1.1–9.5). The effect of sIPSC frequency is 3.9 pA per 1 s^−1^ larger after VU0463271 exposure compared to controls (95% CrI = −1.07–9.03). ACSF, artificial cerebrospinal fluid. sIPSC, spontaneus postsynaptic currents.

Next, we aimed to explore adaptive changes in routes of Cl^−^ membrane passage, alternative to KCC2. We concentrated on tonic inhibition, because it has been shown (1) to rely on high‐affinity GABA_A_R outside the synaptic cleft and to be preserved in neocortical neurons of human TLE patients, despite the internalization of synaptic GABA_A_R^44^; and (2) to partially substitute for the loss of other conductance (such as for instance *I*_h_ ^45^) in the context of epilepsy.^46^ It may further challenge or support KCC2 by acting as a permanent [Cl^−^]_*i*_ source or sink, depending on *E*_Cl_−. Given predominantly positive *DF*_GABA(A)_ in our single channel GABA_A_R recordings, which implies a somatic *E*_Cl_− above the membrane potential (even when taking the HCO_3_^−^‐induced 10–15 mV positive shift into account; see Figure S5 for details), we assumed a prevailing inward (depolarizing) Cl^−^ current. Therefore, we conducted whole‐cell somatic recordings of supragranular pyramidal neurons under [Cl^−^]_*i*_ load (Figure 5A). The large somatic injection of Cl^−^ additionally enabled us to record enhanced currents (to detect small amounts) and to minimize the contribution of HCO_3_^−^ to the GABA_A_R current. Technically, the high [Cl^−^]_*i*_ helped to otherwise reflect the in vivo situation by enabling recordings at near resting membrane potential (at a command voltage of −73 mV) without the need to add either GABA or inhibitors of GABA transport. The bicuculline‐sensitive (GABA_A_R‐mediated) holding current corresponds to the tonic inhibition (Figure 5B,C). Whereas short‐term application, although increasing the holding current, hardly augmented the tonic inhibition (Figure S6), longer block of KCC2, that is, a preincubation period of >2 h with VU0463271, clearly increased it. Addition of bicuculline revealed that the amplitude of tonic currents tripled, and its density doubled after longer KCC2 block (Figure 5B,C). In both cases, with and without KCC2 block, the amount of tonic inhibition depended on the preceding spontaneous presynaptic GABA release. However, KCC2 block increased the sensitivity of tonic inhibition to ambient GABA (Figure 5D).

Therewith, tonic GABA_A_ currents may contribute to the accumulation of [Cl^−^]_*i*_ when [Cl^−^]_*i*_ is low and extrusion capacity is compromised. However, as *DF*_GABA(A)_ putatively favors inward GABA_A_‐mediated Cl^−^ currents in most neurons even if taking the parallel and constantly inward HCO_3_^−^ current into account, tonic GABA_A_R activity may mostly extrude accumulated [Cl^−^]_*i*_.

## 4 DISCUSSION

Against the background of GABAergic transients as one major seizure‐facilitating mechanism,^47^ we substantiated the importance of Cl^−^ extrusion via KCC2 in the seizure propagation zone of humans. It evidently outweighs the consequences of the concomitant [K^+^]_*o*_ increase. The opposing effect of acute attenuating and augmenting pharmacological modulation of KCC2 indicates that KCC2 function dampens occurrence and propagation of ictal‐like network activity in human supragranular neocortex. On the cellular level, artificial [Cl^−^]_*i*_ load overburdens the KCC2 extrusion capacity, predominantly at the soma. The basal KCC2 extrusion capacity may vary, as individual pyramidal neurons of TLE patients have diverging, predominantly depolarizing somatic *DF*_GABA(A)_. These driving forces may enable GABA_A_Rs, which mediate persisting tonic inhibition, to support the weakened Cl^−^ extrusion.

### 4.1 Role of KCC2 in the human seizure propagation zone

Our findings emphasize the relevance of KCC2 dysfunction‐related, depolarizing GABAergic transmission that was previously reported in hippocampal and neocortical neurons from patients suffering from TLE^32^, ^35^, ^48^ or pediatric focal cortical dysplasia.^34^ Because we performed experiments outside the epileptogenic zone, that is, in the neocortical seizure‐propagating supragranular network involved in the continuation and propagation of seizures in vivo,^1^ our results extend the current knowledge of the KCC2 contribution in seizure propagation and putatively constitute a unifying feature of seizures beyond the context of TLE. In line with this, seizure‐facilitating KCC2 dysfunction has been previously demonstrated in peritumoral tissue.^36^

Given the low maximum velocity of KCC2 transport relative to Cl^−^ membrane flux,^49^ the KCC2‐mediated Cl^−^ extrusion becomes crucial as synaptic activity increases and can saturate even in the absence of functional downregulation.^50^ Therefore, synaptic activity leads to a gradual [Cl^−^]_*i*_ buildup^5^, ^49^ when neurons depolarize for a longer period,^51^ that is, at maximum attainable synaptic activity.^52^ [Cl^−^]_*i*_ load could be augmented by coincident glutamatergic depolarization^53^ and by the Na^+^/K^+^/2Cl^−^ cotransporter1 (NKCC1).^54^ Accordingly, GABA_A_R and NKCC1 antagonists decrease spontaneous interictal discharges in acute hippocampal slices from patients with TLE^35^ and focal cortical dysplasia.^34^

The large intrapatient variance of somatodendritic *E*_GABA(A)_ gradients supports the findings of locally varying KCC2 function.^35^ The gradient itself points to differential consequences of Cl^−^ at somatic versus dendritic sites. Due to the differing surface to volume ratio and subcellular KCC2 expression,^19^ somatic [Cl^−^]_*i*_ might remain higher when KCC2 function is impaired. Divergence between [Cl^−^]_*p*_ and the somatic [Cl^−^]_*i*_ derived from the measured *E*_GABA(A)_ indicates somatic KCC2 activity in TLE neurons. However, as [Cl^−^]_*p*_ increases, the difference diminishes, suggesting that somatic Cl^−^ extrusion reaches its limits (Δ [Cl^−^]_*p*_ – somatic [Cl^−^]_*i*_ = 11.1 mmol·L^−1^ Cl^−^, 95% CrI = 12.9–9.3 for 19 mmol·L^−1^, Δ [Cl^−^]_*p*_ – somatic [Cl^−^]_*i*_ = 8.9 mmol·L^−1^ Cl^−^, 95% CrI = 17.4–.3 for 41 mmol·L^−1^). In turn, elevated Cl^−^ would affect perisomatic inhibition^55^ more than dendritic inhibition. This would influence the timing of pyramidal firing that is determined by the activity of parvalbuminergic basket cells^56^ targeting the soma. Parvalbumin‐containing neurons are critically involved in rhythmic synchrony of neocortical networks^57^, ^58^ known to be abnormal in epilepsy. Preferential somatic [Cl^−^]_*i*_ elevations would affect not only parvalbumin but also cholecystokinin inputs and thereby alter the plastic fine‐tuning that modulates neuronal ensemble activities in response to information about motivations, emotions, and the autonomic state, also described as the “mood”^55^ of network oscillations.

Because KCC2 is downregulated in a wide spectrum of pathophysiological conditions ranging from neuropsychiatric disorders to epilepsy,^59^, ^60^ acute KCC2 modulation might occur on top of long‐term alterations due to recurring seizures. Post hoc relation of KCC2‐governed parameters and preoperative seizure frequency yielded an association of daily seizures with a relevant decrease of the somatodendritic *E*_GABA(A)_ gradient at higher Cl^−^ load (41 mmol·L^−1^ [Cl^−^]_*p*_) but not at lower load (19 mmol·L^−1^ [Cl^−^]_*p*_; Figure S7). This suggests that a high seizure burden impairs the neuronal, mainly KCC2‐mediated, capability to extrude Cl^−^ at acute additional Cl^−^ loads (e.g., during a seizure) in the seizure propagation zone and extends findings on reduced KCC2 expression or function, as in human neurons that discharge during epileptic events,^32^, ^35^, ^61^ in neurons of patients suffering from TLE, showing depolarizing GABAergic transmission^62^, ^63^ and reduced KCC2‐mediated (somatic) Cl^−^ extrusion in a subgroup of supragranular neocortical neurons.^64^ The apparent contrast to the reported increase in overall KCC2 expression in brain tissue of patients suffering from TLE^65^ might be solved by the restriction of KCC2 downregulation in epilepsy to specific areas.^34^ Note that our data cannot exclude that decreased KCC2‐mediated Cl^−^ extrusion is the cause rather than the consequence of frequent preoperative seizures.

We interpret the tonic inhibition, which tends to increase with preoperative seizure frequency (Figure S7), as a protective adaptation that partially compensates KCC2 insufficiency during [Cl^−^]_*i*_ load and prevents excessive accumulation of [Cl^−^]_*i*_ following strong GABAergic synaptic activity without further elevating [K^+^]_*o*_. This view is corroborated by our finding that CLP257, a positive allosteric regulator of extrasynaptic GABA_A_R (in addition to being an enhancer of KCC2 clustering and function),^38^, ^39^, ^40^ readily diminished ILEs, and by the association of epilepsy syndromes with loss‐of‐function mutations in extrasynaptic GABA_A_R genes.^66^ Note that tonic inhibition might be pronounced in the majority of our MEA recordings, because 6 mmol·L^−1^ K^+^ has been reported to reverse GAT‐1 function.^67^ Whereas the critical role of tonic inhibition in regulating network excitability^68^ is beyond doubt, the computational consequences for preferentially outward tonic signaling are yet to be determined.

Our data suggest that KCC2 is functional in almost all pyramidal neurons tested of TLE patients, and its acute modulation reciprocally impacts epileptiform activity in the seizure propagation zone. Transient insufficiency of KCC2 could ease seizure propagation and contribute to the limited therapeutic efficacy of conventional antiseizure medications in TLE.^69^, ^70^ Strategies aiming to restore neuronal Cl^−^ ionostasis by stabilizing KCC2 membrane expression,^38^, ^71^ or increasing KCC2 activity by disinhibiting the WNK‐SPAK pathway,^22^ would be beneficial in TLE, because the concomitant [K^+^]_*o*_ increase appears to be transient and/or compensated for.

## AUTHOR CONTRIBUTIONS

*MEA recordings:* Alice Falck. *MEA data analysis:* Alice Falck and Mahraz Behbood. *Algorithm and pipeline MEA data analysis:* Mahraz Behbood, Alice Falck, Jan‐Hendrik Schleimer, and Susanne Schreiber. *Patch‐clamp recording and analysis:* Janna Lehnhoff, Egor Byvaltcev, Noah Döhne, Gabriel M. S. Janach, Alice Falck, and Ulf Strauss. *Statistical analysis:* Alice Falck and Annette Aigner. *Patient material:* Pawel Fidzinski, Martin Holtkamp, Julia Onken, Thilo Kalbhenn, and Thomas Sauvigny. *Neuropathological assessment:* Helena Radbruch. *Cowrote first draft:* Alice Falck, Janna Lehnhoff, and Ulf Strauss. *Manuscript editing:* all authors. *Design and conception of the study:* Ulf Strauss, Alice Falck, and Rudolf A. Deisz.

## CONFLICT OF INTEREST STATEMENT

None of the authors has any conflict of interest to disclose. We confirm that we have read the Journal's position on issues involved in ethical publication and affirm that this report is consistent with those guidelines.

## Supporting information


Data S1.

## Data Availability

The MEA analysis pipeline is available online (zenodo platform at DOI: 10.5281/zenodo.15802113). Data are available from the corresponding author upon request.

## References

[1] Bourdillon P , Ren L , Halgren M , Paulk AC , Salami P , Ulbert I , et al. Differential cortical layer engagement during seizure initiation and spread in humans. Nat Commun. 2024;15:5153. 38886376 10.1038/s41467-024-48746-8 PMC11183216

[2] Gastaut H , Broughton RJ . Epileptic seizures; clinical and electrographic features, diagnosis and treatment. Springfield, IL: Thomas; 1972.

[3] Trevelyan AJ , Sussillo D , Watson BO , Yuste R . Modular propagation of epileptiform activity: evidence for an inhibitory veto in neocortex. J Neurosci. 2006;26:12447–12455. 17135406 10.1523/JNEUROSCI.2787-06.2006 PMC6674895

[4] Kaila K , Trevelyan A , Raimondo J , Ala‐Kurikka T , Huberfeld G , Avoli M , et al. GABAA‐receptor signaling and ionic plasticity in the generation and spread of seizures. Jasper's basic mechanisms of the epilepsies. New York: Oxford University Press; 2024. 10.1093/med/9780197549469.003.0006 39637123

[5] Staley KJ , Proctor WR . Modulation of mammalian dendritic GABA(A) receptor function by the kinetics of Cl‐ and HCO3‐ transport. J Physiol. 1999;519:693–712. 10457084 10.1111/j.1469-7793.1999.0693n.x PMC2269533

[6] Prince DA , Connors BW . Mechanisms of epileptogenesis in cortical structures. Ann Neurol. 1984;16:S59–S64. 6095743 10.1002/ana.410160710

[7] Kaila K . Ionic basis of GABAA receptor channel function in the nervous system. Prog Neurobiol. 1994;42:489–537. 7522334 10.1016/0301-0082(94)90049-3

[8] Kaila K , Voipio J . Postsynaptic fall in intracellular pH induced by GABA‐activated bicarbonate conductance. Nature. 1987;330:163–165. 3670401 10.1038/330163a0

[9] Kaila K , Lamsa K , Smirnov S , Taira T , Voipio J . Long‐lasting GABA‐mediated depolarization evoked by high‐frequency stimulation in pyramidal neurons of rat hippocampal slice is attributable to a network‐driven, bicarbonate‐dependent K transient. J Neurosci. 1997;17:7662–7672. 9315888 10.1523/JNEUROSCI.17-20-07662.1997 PMC6793904

[10] Kaila K , Voipio J , Paalasmaa P , Pasternack M , Deisz RA . The role of bicarbonate in GABAA receptor‐mediated IPSPs of rat neocortical neurones. J Physiol. 1993;464:273–289. 8229801 10.1113/jphysiol.1993.sp019634 PMC1175385

[11] Jarolimek W , Lewen A , Misgeld U . A furosemide‐sensitive K+‐Cl− cotransporter counteracts intracellular Cl− accumulation and depletion in cultured rat midbrain neurons. J Neurosci. 1999;19:4695–4704. 10366603 10.1523/JNEUROSCI.19-12-04695.1999 PMC6782681

[12] Payne JA , Stevenson TJ , Donaldson LF . Molecular characterization of a putative K‐Cl cotransporter in rat brain: a neuronal‐specific isoform. J Biol Chem. 1996;271:16245–16252. 8663311 10.1074/jbc.271.27.16245

[13] Sivakumaran S , Cardarelli RA , Maguire J , Kelley MR , Silayeva L , Morrow DH , et al. Selective inhibition of KCC2 leads to hyperexcitability and epileptiform discharges in hippocampal slices and in vivo. J Neurosci. 2015;35:8291–8296. 26019342 10.1523/JNEUROSCI.5205-14.2015 PMC4444547

[14] Kelley MR , Deeb TZ , Brandon NJ , Dunlop J , Davies PA , Moss SJ . Compromising KCC2 transporter activity enhances the development of continuous seizure activity. Neuropharmacology. 2016;108:103–110. 27108931 10.1016/j.neuropharm.2016.04.029 PMC5337122

[15] Dzhala VI , Staley KJ . Kcc2 chloride transport contributes to the termination of ictal epileptiform activity. eNeuro. 2021;8:ENEURO.0208–ENEU20.2020. 10.1523/ENEURO.0208-20.2020 PMC7986536 33239270

[16] Moore YE , Deeb TZ , Chadchankar H , Brandon NJ , Moss SJ . Potentiating KCC2 activity is sufficient to limit the onset and severity of seizures. Proc Natl Acad Sci USA. 2018;115:10166–10171. 30224498 10.1073/pnas.1810134115 PMC6176565

[17] Viitanen T , Ruusuvuori E , Kaila K , Voipio J . The K+‐Cl‐ cotransporter KCC2 promotes GABAergic excitation in the mature rat hippocampus. J Physiol. 2010;588:1527–1540. 20211979 10.1113/jphysiol.2009.181826 PMC2876807

[18] Byvaltcev E , Behbood M , Schleimer JH , Gensch T , Semyanov A , Schreiber S , et al. KCC2 reverse mode helps to clear postsynaptically released potassium at glutamatergic synapses. Cell Rep. 2023;42:112934. 37537840 10.1016/j.celrep.2023.112934 PMC10480490

[19] Weilinger NL , Wicki‐Stordeur LE , Groten CJ , LeDue JM , Kahle KT , MacVicar BA . KCC2 drives chloride microdomain formation in dendritic blebbing. Cell Rep. 2022;41:111556. 36288701 10.1016/j.celrep.2022.111556

[20] Hamidi S , Avoli M . KCC2 function modulates in vitro ictogenesis. Neurobiol Dis. 2015;79:51–58. 25926348 10.1016/j.nbd.2015.04.006 PMC4880462

[21] González OC , Shiri Z , Krishnan GP , Myers TL , Williams S , Avoli M , et al. Role of KCC2‐dependent potassium efflux in 4‐aminopyridine‐induced epileptiform synchronization. Neurobiol Dis. 2018;109:137–147. 29045814 10.1016/j.nbd.2017.10.011 PMC5710807

[22] Lee KL , Abiraman K , Lucaj C , Ollerhead TA , Brandon NJ , Deeb TZ , et al. Inhibiting with‐no‐lysine kinases enhances K+/Cl− cotransporter 2 activity and limits status epilepticus. Brain. 2022;145:950–963. 34528073 10.1093/brain/awab343 PMC9050525

[23] Lehnhoff J , Strauss U , Wierschke S , Grosser S , Pollali E , Schneider UC , et al. The anticonvulsant lamotrigine enhances I h in layer 2/3 neocortical pyramidal neurons of patients with pharmacoresistant epilepsy. Neuropharmacology. 2019;144:58–69. 30315843 10.1016/j.neuropharm.2018.10.004

[24] Eichele T , Calhoun VD , Debener S . Mining EEG–fMRI using independent component analysis. Int J Psychophysiol. 2009;73:53–61. 19223007 10.1016/j.ijpsycho.2008.12.018 PMC2693483

[25] Cotterill E , Eglen SJ . Burst detection methods in vitro neuronal networks. In: Chiappalone M , Pasquale V , Frega M , editors. Advances in Neurobiology: In Vitro Neuronal Networks. Volume 22. Switzerland AG: Springer Nature; 2019. p. 185–206. 10.1007/978-3-030-11135-9_8 31073937

[26] Farrant M , Kaila K . The cellular, molecular and ionic basis of GABAA receptor signalling. Prog Brain Res. 2007;160:59–87. 10.1016/S0079-6123(06)60005-8 17499109

[27] Lazic SE . The problem of pseudoreplication in neuroscientific studies: is it affecting your analysis? BMC Neurosci. 2010;11:1–17. 10.1186/1471-2202-11-5 PMC2817684 20074371

[28] Amrhein V , Greenland S , McShane B . Scientists rise up against statistical significance. Nature. 2019;567:305–307. 30894741 10.1038/d41586-019-00857-9

[29] Palesch YY . Some common misperceptions about p values. Stroke. 2014;45:1–7. 10.1161/STROKEAHA.114.006138 PMC4245357 25378423

[30] Schevon CA , Weiss SA , McKhann G Jr , Goodman RR , Yuste R , Emerson RG , et al. Evidence of an inhibitory restraint of seizure activity in humans. Nat Commun. 2012;3:1060. 22968706 10.1038/ncomms2056 PMC3658011

[31] Spencer SS , Guimaraes P , Katz A , Kim J , Spencer D . Morphological patterns of seizures recorded intracranially. Epilepsia. 1992;33:537–545. 1592034 10.1111/j.1528-1157.1992.tb01706.x

[32] Cohen I , Navarro V , Clemenceau S , Baulac M , Miles R . On the origin of interictal activity in human temporal lobe epilepsy in vitro. Science. 2002;1979(298):1418–1421. 10.1126/science.1076510 12434059

[33] Huberfeld G , Menendez de la Prida L , Pallud J , Cohen I , le van Quyen M , Adam C , et al. Glutamatergic pre‐ictal discharges emerge at the transition to seizure in human epilepsy. Nat Neurosci. 2011;14:627–634. 21460834 10.1038/nn.2790

[34] Blauwblomme T , Dossi E , Pellegrino C , Goubert E , Iglesias BG , Sainte‐Rose C , et al. Gamma‐aminobutyric acidergic transmission underlies interictal epileptogenicity in pediatric focal cortical dysplasia. Ann Neurol. 2019;85:204–217. 30597612 10.1002/ana.25403

[35] Huberfeld G , Wittner L , Clemenceau S , Baulac M , Kaila K , Miles R , et al. Perturbed chloride homeostasis and GABAergic signaling in human temporal lobe epilepsy. J Neurosci. 2007;27:9866–9873. 17855601 10.1523/JNEUROSCI.2761-07.2007 PMC6672644

[36] Pallud J , le van Quyen M , Bielle F , Pellegrino C , Varlet P , Labussiere M , et al. Cortical GABAergic excitation contributes to epileptic activities around human glioma. Sci Transl Med. 2014;6:244ra89. 10.1126/scitranslmed.3008065 PMC4409113 25009229

[37] Delpire E , Baranczak A , Waterson AG , Kim K , Kett N , Morrison RD , et al. Further optimization of the K‐Cl cotransporter KCC2 antagonist ML077: development of a highly selective and more potent in vitro probe. Bioorg Med Chem Lett. 2012;22:4532–4535. 22727639 10.1016/j.bmcl.2012.05.126 PMC3389279

[38] Gagnon M , Bergeron MJ , Lavertu G , Castonguay A , Tripathy S , Bonin RP , et al. Chloride extrusion enhancers as novel therapeutics for neurological diseases. Nat Med. 2013;19:1524–1528. 24097188 10.1038/nm.3356 PMC4005788

[39] Cardarelli RA , Jones K , Pisella LI , Wobst HJ , McWilliams L , Sharpe PM , et al. The small molecule CLP257 does not modify activity of the K+–Cl^−^ co‐transporter KCC2 but does potentiate GABA A receptor activity. Nat Med. 2017;23:1394–1396. 10.1038/nm.4442 29216042 PMC7371006

[40] Donneger F , Zanin A , Besson J , Kadiri Y , Pagan C , David N , et al. Enhancing KCC2 function reduces interictal activity and prevents seizures in mesial temporal lobe epilepsy. Bio Rxiv. 2023;1–32. 10.1073/pnas.2522722123 PMC12974483 41774803

[41] Heubl M , Zhang J , Pressey JC , Al Awabdh S , Renner M , Gomez‐Castro F , et al. GABAA receptor dependent synaptic inhibition rapidly tunes KCC2 activity via the Cl^−^‐sensitive WNK1 kinase. Nat Commun. 2017;8:1776. 29176664 10.1038/s41467-017-01749-0 PMC5701213

[42] Kadam SD , Hegarty SV . Development of KCC2 therapeutics to treat neurological disorders. Front Mol Neurosci. 2024;17:1503070. 39720463 10.3389/fnmol.2024.1503070 PMC11666659

[43] Kurki SN , Srinivasan R , Laine J , Virtanen MA , Ala‐Kurikka T , Voipio J , et al. Acute neuroinflammation leads to disruption of neuronal chloride regulation and consequent hyperexcitability in the dentate gyrus. Cell Rep. 2023;42:113379. 37922309 10.1016/j.celrep.2023.113379

[44] Scimemi A , Andersson A , Heeroma JH , Strandberg J , Rydenhag B , McEvoy AW , et al. Tonic GABAA receptor‐mediated currents in human brain. Eur J Neurosci. 2006;24:1157–1160. 16930441 10.1111/j.1460-9568.2006.04989.x

[45] Strauss U , Kole MHP , Bräuer AU , Pahnke J , Bajorat R , Rolfs A , et al. An impaired neocortical Ih is associated with enhanced excitability and absence epilepsy. Eur J Neurosci. 2004;19:3048–3058. 15182313 10.1111/j.0953-816X.2004.03392.x

[46] Chen X , Shu S , Schwartz LC , Sun C , Kapur J , Bayliss DA . Homeostatic regulation of synaptic excitability: tonic GABAA receptor currents replace Ih in cortical pyramidal neurons of HCN1 knock‐out mice. J Neurosci. 2010;30:2611–2622. 20164346 10.1523/JNEUROSCI.3771-09.2010 PMC2830721

[47] Löscher W , Puskarjov M , Kaila K . Cation‐chloride cotransporters NKCC1 and KCC2 as potential targets for novel antiepileptic and antiepileptogenic treatments. Neuropharmacology. 2013;69:62–74. 10.1016/j.neuropharm.2012.05.045 22705273

[48] Deisz RA , Lehmann TN , Horn P , Dehnicke C , Nitsch R . Components of neuronal chloride transport in rat and human neocortex. J Physiol. 2011;589:1317–1347. 21224237 10.1113/jphysiol.2010.201830 PMC3082095

[49] Doyon N , Vinay L , Prescott SA , De Koninck Y . Chloride regulation: a dynamic equilibrium crucial for synaptic inhibition. Neuron. 2016;89:1157–1172. 10.1016/j.neuron.2016.02.030 26985723

[50] Kahle KT , Deeb TZ , Puskarjov M , Silayeva L , Liang B , Kaila K , et al. Modulation of neuronal activity by phosphorylation of the K‐Cl cotransporter KCC2. Trends Neurosci. 2013;36:726–737. 10.1016/j.tins.2013.08.006 24139641 PMC4381966

[51] Rungta RL , Choi HB , Tyson JR , Malik A , Dissing‐Olesen L , Lin PJC , et al. The cellular mechanisms of neuronal swelling underlying cytotoxic edema. Cell. 2015;161:610–621. 25910210 10.1016/j.cell.2015.03.029

[52] Alfonsa H , Merricks EM , Codadu NK , Cunningham MO , Deisseroth K , Racca C , et al. The contribution of raised intraneuronal chloride to epileptic network activity. J Neurosci. 2015;35:7715–7726. 25995461 10.1523/JNEUROSCI.4105-14.2015 PMC4438123

[53] Currin CB , Trevelyan AJ , Akerman CJ , Raimondo JV . Chloride dynamics alter the input‐output properties of neurons. PLoS Comput Biol. 2020;16:e1007932. 32453795 10.1371/journal.pcbi.1007932 PMC7307785

[54] Delpire E , Rauchman MI , Beier DR , Hebert SC , Gullans SR . Molecular cloning and chromosome localization of a putative basolateral Na+‐K+‐2Cl‐ cotransporter from mouse inner medullary collecting duct (mIMCD‐3) cells. J Biol Chem. 1994;269:25677–25683. 7929272

[55] Freund TF , Katona I . Perisomatic inhibition. Neuron. 2007;56:33–42. 10.1016/j.neuron.2007.09.012 17920013

[56] Cobb SR , Buhl EH , Halasy K , Paulsen O , Somogyi P . Synchronization of neuronal activity in hippocampus by individual GABAergic interneurons. Nature. 1995;378:75–78. 7477292 10.1038/378075a0

[57] Cossart R , Ikegaya Y , Yuste R . Calcium imaging of cortical networks dynamics. Cell Calcium. 2005;37:451–457. 15820393 10.1016/j.ceca.2005.01.013

[58] Ogiwara I , Miyamoto H , Morita N , Atapour N , Mazaki E , Inoue I , et al. Nav1.1 localizes to axons of parvalbumin‐positive inhibitory interneurons: a circuit basis for epileptic seizures in mice carrying an Scn1a gene mutation. J Neurosci. 2007;27:5903–5914. 17537961 10.1523/JNEUROSCI.5270-06.2007 PMC6672241

[59] Kaila K , Price TJ , Payne JA , Puskarjov M , Voipio J . Cation‐chloride cotransporters in neuronal development, plasticity and disease. Nat Rev Neurosci. 2014;15:637–654. 25234263 10.1038/nrn3819 PMC4294553

[60] Moore YE , Kelley MR , Brandon NJ , Deeb TZ , Moss SJ . Seizing control of KCC2: a new therapeutic target for epilepsy. Trends Neurosci. 2017;40:555–571. 28803659 10.1016/j.tins.2017.06.008

[61] Palma E , Amici M , Sobrero F , Spinelli G , di Angelantonio S , Ragozzino D , et al. Anomalous levels of Cl‐ transporters in the hippocampal subiculum from temporal lobe epilepsy patients make GABA excitatory. Proc Natl Acad Sci USA. 2006;103:8465–8468. 16709666 10.1073/pnas.0602979103 PMC1482515

[62] Huberfeld G , Blauwblomme T , Miles R . Hippocampus and epilepsy: findings from human tissues Europe PMC Funders Group. Rev Neurol (Paris). 2015;171:236–251. 25724711 10.1016/j.neurol.2015.01.563 PMC4409112

[63] Miles R , Blaesse P , Huberfeld G , Wittner L , Kaila K . Chloride homeostasis and GABA signaling in temporal lobe epilepsy. In: Noebels JL , Avoli M , Rogawski MA , Olsen RW , Delgado‐Escueta AV , editors. Jasper's Basic Mechanisms of the Epilepsies. 4th ed. Bethesda (MD): National Center for Biotechnology information (US); 2012.p. 675–686. 22787654

[64] Deisz RA , Wierschke S , Schneider UC , Dehnicke C . Effects of VU0240551, a novel KCC2 antagonist, and DIDS on chloride homeostasis of neocortical neurons from rats and humans. Neuroscience. 2014;277:831–841. 25086309 10.1016/j.neuroscience.2014.07.037

[65] Karlócai MR , Wittner L , Tóth K , Maglóczky Z , Katarova Z , Rásonyi G , et al. Enhanced expression of potassium‐chloride cotransporter KCC2 in human temporal lobe epilepsy. Brain Struct Funct. 2016;221:3601–3615. 26427846 10.1007/s00429-015-1122-8

[66] Mulley JC , Scheffer IE , Harkin LA , Berkovic SF , Dibbens LM . Susceptibility genes for complex epilepsy. Hum Mol Genet. 2005;14:243–249. 10.1093/hmg/ddi355 16244322

[67] Wu Y , Wang W , Richerson GB . GABA transaminase inhibition induces spontaneous and enhances depolarization‐evoked GABA efflux via reversal of the GABA transporter. J Neurosci. 2001;21:2630–2639. 11306616 10.1523/JNEUROSCI.21-08-02630.2001 PMC6762542

[68] Semyanov A , Walker MC , Kullmann DM . GABA uptake regulates cortical excitability via cell type‐specific tonic inhibition. Nat Neurosci. 2003;6:484–490. 12679782 10.1038/nn1043

[69] Stephen LJ , Kwan P , Brodie MJ . Does the cause of localisation‐related epilepsy influence the response to antiepileptic drug treatment? Epilepsia. 2001;42:357–362. 11442153 10.1046/j.1528-1157.2001.29000.x

[70] Semah F , Picot MC , Adam C , Broglin D , Arzimanoglou A , Bazin B , et al. Is the underlying cause of epilepsy a major prognostic factor for recurrence? Neurology. 1998;51:1256–1262. 9818842 10.1212/wnl.51.5.1256

[71] Liabeuf S , Stuhl‐Gourmand L , Gackière F , Mancuso R , Sanchez Brualla I , Marino P , et al. Prochlorperazine increases KCC2 function and reduces spasticity after spinal cord injury. J Neurotrauma. 2017;34:3397–3406. 28747093 10.1089/neu.2017.5152

