# Supplementary material for: Potassium–chloride cotransporter 2 activity dampens induced ictal‐like activity in neocortical slices containing the seizure propagation zone of temporal lobe epilepsy patients
Source: Epilepsia. 2025 Sep 11;67(1):499–516. doi: 10.1111/epi.18630 (PMC12893269; doi:10.1111/epi.18630)
Supplement: Supplementary file 1 — Data S1. [file EPI-67-499-s001.pdf]

Supplementary Material

Table S1: Solutions

Solution	Composition in mM	pH	Osmolarity in mosmol/l
**Extracellular solution**			
ACSF	NaCl 119, KCl 2.5, NaH_2PO_4 1, $NaHCO_3$ 26, $CaCl_2$ 2.5, $MgCl_2$ 1.3, Glucose 10	7.4	290-300
0 mM Mg^{2+} modified ACSF	NaCl 119, KCl 6, NaH_2PO_4 1, $NaHCO_3$ 26, $CaCl_2$ 2.5, Glucose 10	7.4	290-300
0.25 mM Mg^{2+} modified ACSF	NaCl 119, KCl 6, NaH_2PO_4 1, $NaHCO_3$ 26, $CaCl_2$ 1, $MgCl_2$ 0.25, Glucose 10	7.4	290-300
0 mM Mg^{2+}, 3 mM K^+ modified ACSF	NaCl 119, KCl 3, NaH_2PO_4 1, $NaHCO_3$ 26, $CaCl_2$ 1, Glucose 10	7.4	290-300
sACSF	NaCl 85, KCl 2.5, NaH_2PO_4 1, $NaHCO_3$ 26, $CaCl_2$ 0.5, $MgCl_2$ 7, Glucose 10, Sucrose 50	7.4	290-300
hACSF	NaCl 92, KCl 2.5, NaH_2PO_4 1.2, $NaHCO_3$ 30, $CaCl_2$ 2, $MgSO_4$ 2, HEPES 20, Sodium ascorbate 5, Sodium pyruvate 3, Thiourea 2, Glucose 25	7.4	290-300
**Pipette solution**			
Whole cell recordings 19 mM Cl^-	NaCl 4, $MgCl_2$ 2, $CaCl_2$ 1, HEPES-K 10, EGTA 11, MgATP 2, GTP 0.5, K-gluconate 117, KCl 9	7.2	295-305
Whole cell recordings 41 mM Cl^-	NaCl 4, $MgCl_2$ 2, $CaCl_2$ 1, HEPES-K 10, EGTA 11, MgATP 2, GTP 0.5 and K-gluconate 95 and KCl 31	7.2	295-305
Cell-attached recordings	NaCl 120, KCl 5, $MgCl_2$ 10, $CaCl_2$ 0.5, HEPES 10, Glucose 10, TEA 20, 4-AP 5 + GABA (1-5 µM)	7.2	315-325
Tonic inhibition recordings	CsCl 149, NaCl 4, $MgCl_2$ 1, HEPES 10, EGTA 11, GTP 0.3, MgATP 2 and QX-314 5	7.2	295-305

Extracellular solutions were carbonated with 95 % O2; 5 % CO_2; ACSF, artificial cerebrospinal fluid; sACSF, sACSF, sucrose-containing artificial cerebrospinal fluid; hACSF, HEPES-containing artificial cerebrospinal fluid.

Table S2: Chemicals

Reagent	Source	Identifier
(–)-Bicuculline methiodide	Tocris, Bristol, UK	CAS 40709-69-1
VU0463271	Tocris, Bristol, UK	CAS 1391737-01-1
CLP257	Tocris, Bristol, UK	CAS 1181081-71-9
Closantel	Sigma-Aldrich, Steinheim, Germany	CAS 57808-65-8
CNQX disodium salt	Tocris, Bristol, UK	CAS 479347-85-8
DAP-5	Tocris, Bristol, UK	CAS 79055-68-8
GABA	Merck Darmstadt, Germany	CAS 56-12-2
QX-314	Merck Darmstadt, Germany	CAS 21306-56-9
TEA	Sigma-Aldrich, Steinheim, Germany	CAS T2265-100G
4–AP	Sigma-Aldrich, Steinheim, Germany	CAS 504-24-5

Table S3: Specifications of the Bayesian linear mixed effects models

Experiment	Dependent variable	Model formula	Effect(s) of interest
MEA KCC2 blocker	v_{ij1}… network activity parameter before pharmacologic KCC2 manipulation<br>v_{ij0}… network activity parameter under pharmacologic KCC2 manipulation	$(v_{ij1} - v_{ij0}) = \beta_0 + u_{0j} + \varepsilon_{ij}$<br>$(v_{ij1} - v_{ij0}) = \beta_0 + \beta_1 * 1_{\{y_{ij0}=0\}} + u_{0j} + \varepsilon_{ij}$	β_0<br>β_0, $(\beta_0 + \beta_1)$
MEA KCC2 enhancer		$(v_{ij1} - v_{ij0}) = \beta_0 + \beta_1 * \overline{y_{ij0}} + u_{0j} + \varepsilon_{ij}$	β_0, β_1
KCC2 extrusion capacity	w_{ij0}… $E_{GABA(A)}$ at soma<br>w_{ij1}… $E_{GABA(A)}$ at dendrite	$(w_{ij0} - w_{ij1}) = \beta_0 + u_{0j} + \varepsilon_{ij}$<br>$(w_{ij0} - w_{ij1}) = \beta_0 + \beta_1 * chloride\ load_{ij} + u_{0j} + \varepsilon_{ij}$<br>$w_{ij0} = \beta_0 + \beta_1 * chloride\ load_{ij} + u_{0j} + \varepsilon_{ij}$<br>$w_{ij1} = \beta_0 + \beta_1 * chloride\ load_{ij} + u_{0j} + \varepsilon_{ij}$	β_0<br>β_1<br>β_1<br>β_1
GABA(A) driving force	x_{ij}… $GABA_A$ driving force and $GABA_A$ conductance<br>x_{ij}… $GABA_A$ driving force and $GABA_A$ conductance	$x_{ij} = \beta_0 + u_{0j} + \varepsilon_{ij}$<br>$x_{ij} = \beta_0 + \beta_1 * Group_{ij} + u_{0j} + \varepsilon_{ij}$	β_0<br>β_1
Tonic inhibition	y_{ij0}… holding current before BMI<br>y_{ij1}… holding current under BMI	$(y_{ij1} - y_{ij0}) = \beta_0 + \beta_1 * substance_{ij} + u_{0j} + \varepsilon_{ij}$<br>$(y_{ij1} - y_{ij0}) = \beta_0 + \beta_1 * sEPSC\ frequency_{ij} + \beta_2 * substance_{ij} + \beta_3 * substance_{ij} * sEPSC\ frequency_{ij} + u_{0j} + \varepsilon_{ij}$	β_1<br>β_3
Influence of seizure frequency	*as defined above*	$(v_{ij1} - v_{ij0}) = \beta_0 + \beta_1 * 1_{\{seizure\ frequency_{ij}=30\}} + u_{0j} + \varepsilon_{ij}$<br>$(w_{ij1} - w_{ij0}) = \beta_0 + \beta_1 * 1_{\{seizure\ frequency_{ij}=30\}} + u_{0j} + \varepsilon_{ij}$<br>$x_{ij} = \beta_0 + \beta_1 * 1_{\{seizure\ frequency_{ij}=30\}} + u_{0j} + \varepsilon_{ij}$<br>$(z_{ij1} - z_{ij0}) = \beta_0 + \beta_1 * 1_{\{seizure\ frequency_{ij}=30\}} + u_{0j} + \varepsilon_{ij}$	β_1<br>β_1<br>β_1<br>β_1

Where i … observation/measurement, j … patient, β_1 … effect of the respective independent variable on the respective dependent variable, u_{0j} … random intercept of patient j, where $u_{0j} \sim N(0, \tau_{00})$, ε_{ij} … residual error for observation/measurement i in patient j.

Supplementary methods

Analysis Pipeline for Microelectrode Array (MEA) Recordings

Data were pre-processed by down-sampling to 200 Hz and low-pass filtering at 50 Hz. Note that in the MEA experiments conducted in ACSF with 0.5 mM Mg^{2+}, 6 mM K^{+}, 1 mM Ca^{2+}, and 0 mM Mg^{2+}, 3 mM K^{+}, 1 mM Ca^{2+}, better results were obtained using a 2–100 Hz bandpass filter, which was therefore applied to this subset. Signals from malfunctioning electrodes that recorded clear artifacts, but not biological activity, were omitted. A source separation pipeline followed preprocessing. Principal component analysis (PCA) was used to select the most relevant principal components (PCs), explaining most of the variance in the data. The number of PCs selected varied depending on the slice and the number of detectable sources of activity. For example, in a slice with two sources of activity, two or three PCs were typically chosen. In cases with very clear signals, two PCs were sufficient. However, if the data were noisy, we included additional PCs representing noise. This approach allowed the noise to be captured and isolated in separate components during the subsequent independent component analysis (ICA). Selected PCs were scaled to unit variance and fed into the fast ICA algorithm.[1] Each resulting independent component (IC) captures one source of induced activity. Field potentials (FP) of ICs were then identified by threshold-based detection (5x RMS (root-mean-square)) and minimum peak prominence of one-fifth of the highest peak. To avoid double-counting, only positive or negative peaks were considered. To detect ictal-like events (ILEs), a burst detection algorithm was applied as follows: ILEs were defined as a minimum of 6 consecutive FPs, with a maximum inter-field-potential interval (IFPI) of 1.5 s. The maximum IFPI was taken from the valley of a bimodal cumulative histogram distribution of all IFPI values across 20 representative recordings (Figure S1C). To capture the slower synchronized discharges at the end of the ILEs, FPs pre- and post-ILE with a maximum IFPI of 4 s were added to each detected ILE. By analyzing the mixing matrix of the ICA (heat maps of absolute ICA weights), electrodes significantly involved in recording the source were identified. Electrodes were considered significantly involved in recording the source if their contribution to the IC reached at least 20%–50% of the maximum contribution observed across all channels (threshold varied depending on the recording). First, the electrode with the highest ICA weight (i.e. the strongest contribution to the IC) was identified. Other electrodes with ICA weights equal to or greater than 20%–50% of this maximum were then classified as involved in recording the source. The same cutoff was used for corresponding recordings before and under pharmacologic treatment. To avoid ILE overestimation, only ILEs of independent sources were counted as separate ILEs. Independence was assumed if ILEs of sources were temporally separated (which can be identified directly by our source separation method), or the direction of activity propagation changed within an experiment. In some slices, synchronous field potentials were observed across multiple electrodes that did not meet the ILE criteria. This was evaluated using the ILE detection algorithm, which confirmed the absence of ILEs. Due to their synchronous nature, compared to spikes/MUA on single electrodes, the field potentials were termed interictal-like discharges (IILDs). The median discharge frequency within ILEs and the mean frequency of IILDs were calculated and reported.

Induction of ictal activity

Intense neuronal activity, that involve NMDA activation including 0 Mg^{2+} epileptiform discharges,[2] has been shown to (temporarily) downregulate KCC2 via NMDA/Ca^{2+}.[3–6] However, several lines of evidence argue against a major effect of low Mg^{2+} on KCC2 for instance via calpain (or – more general – Ca^{2+} influx) during our acute experiments: 1. Time. The 0 Mg^{2+} induced KCC2 reduction became only significant after 4 h (although direct calpain exposure to the brain homogenate reduced KCC2 after 30 min to nearly 0).[4] Our slices were usually exposed to the 0 Mg^{2+} ACSF around 70 min (in sum, i.e. around 40 min pre-exposure and 30 min VU0463271) and even after longer pre-exposure time (60 to 80 min) the effect of VU0463271 developed with the

same kinetic as after the "usual" pre-exposure (Figure S2). 2. Amount (reduction of KCC2 protein to 77% after 4 h 0 Mg^{2+} - maybe because not all neurons recruited to ILE) and 3. Washout experiments. When washing out VU0463271 (still in the presence of the 0 Mg^{2+} ACSF) we saw a profound reduction of ILEs. As an additional indirect hint might be seen that lost efficiency (i.e. the Cl^- gradient broke down) occurres only after the appearance of late recurrent discharges (LRDs).[7] Notably, during our experiments, we never saw the "classical" 0 Mg^{2+}-induced LRDs. We hold the view that the changes following our pharmacological manipulations occur on top of the changes resulting from our low Mg^{2+} solutions. However, we cannot distinguish whether our finding that the KCC2 block is less effective when low Mg^{2+} induced high ILE activity points to (i) an inherent KCC2 dysfunction causing increased ictal-like activity due to low Mg^{2+} or (ii) to a strong low Mg^{2+} induced diminution of KCC2 before the block attempt, or (iii) if both factors contribute.

In addition, previous research suggests that the ictal discharge pattern did not depend on the convulsant used,[8] insinuating that properties of the slice rather than of the specific ACSF used dictate the ictal phenotype. Human tissue appears more resilient than rodent tissue to the induction of ILEs by 0 Mg^{2+}, as was also previously shown in TLE subiculum,[8] where ILEs could be induced in "only" 78% of slices in 79% of patients and exclusively by combining two pro-convulsant stimuli. In that study (as in ours, compare Figure S2), treatments with single convulsants, including 0 Mg^{2+}, were ineffective. Of note in this context, all known methods to induce ILEs could be read as potential inhibitors of KCC2 function, i.e. could be taken as additional hint of the link between KCC2 and ILEs.

Enhancement of KCC2 activity

Although there might be not much evidence of neuronal NKCC1 contribution in "healthy" tissue, since neurons express exceptionally low amounts of NKCC1 (main splice isoform: NKCC1b)[9], we cannot exclude an upregulation of neuronal NKCC1 as suggested in peritumoral tissue[10,11] and in neuronal development.[12,13] However, although reducing neuronal NKCC1 function could dampen ILEs by augmenting the neuronal Cl^- load, it could also turn astrocytes into some kind of Cl^- sink, i.e., interfere with the pivotal seizure protective role of astrocytic NKCC1[14] and increase ILEs. That would counteract the KCC2 effect of closantel and explain inferior efficiency.

Statistical analysis

The presented dataset includes both independent measurements (from different patients) and dependent measurements (multiple recordings from the same patient). Treating multiple recordings from the same patient as independent observations (as done in e.g. paired t-tests) would lead to an incorrect statistical inference, as it ignores the within-subject correlation structure and inflates the effective sample size.[15–17] To address this, Bayesian linear mixed-effects models with random intercepts were used to provide a robust framework for analyzing the hierarchical data structure. Models were fitted with default priors, and 8 chains, each with 4'000 iterations. The mixing of chains and the posterior distribution were visually evaluated. All models converged (Rhat = 1) without divergent transitions. Model assumptions were graphically evaluated based on residual Q-Q plots, histograms, and boxplots, and deemed adequate in the final analyses. Formulas of all fitted models are listed in Table S3. All statistical analyses were conducted using R version 4.3.3.,[18] just as further R packages "brms",[19] "rmcorr",[20] "ggplot2",[21] "patchwork",[22] and "bayestestR".[23] Additionally, OriginPro 2019 (OriginLab Corporation, Northampton, MA, USA) and Affinity Publisher 2 (Serif (Europe) Ltd., Nottingham, United Kingdom) were used.

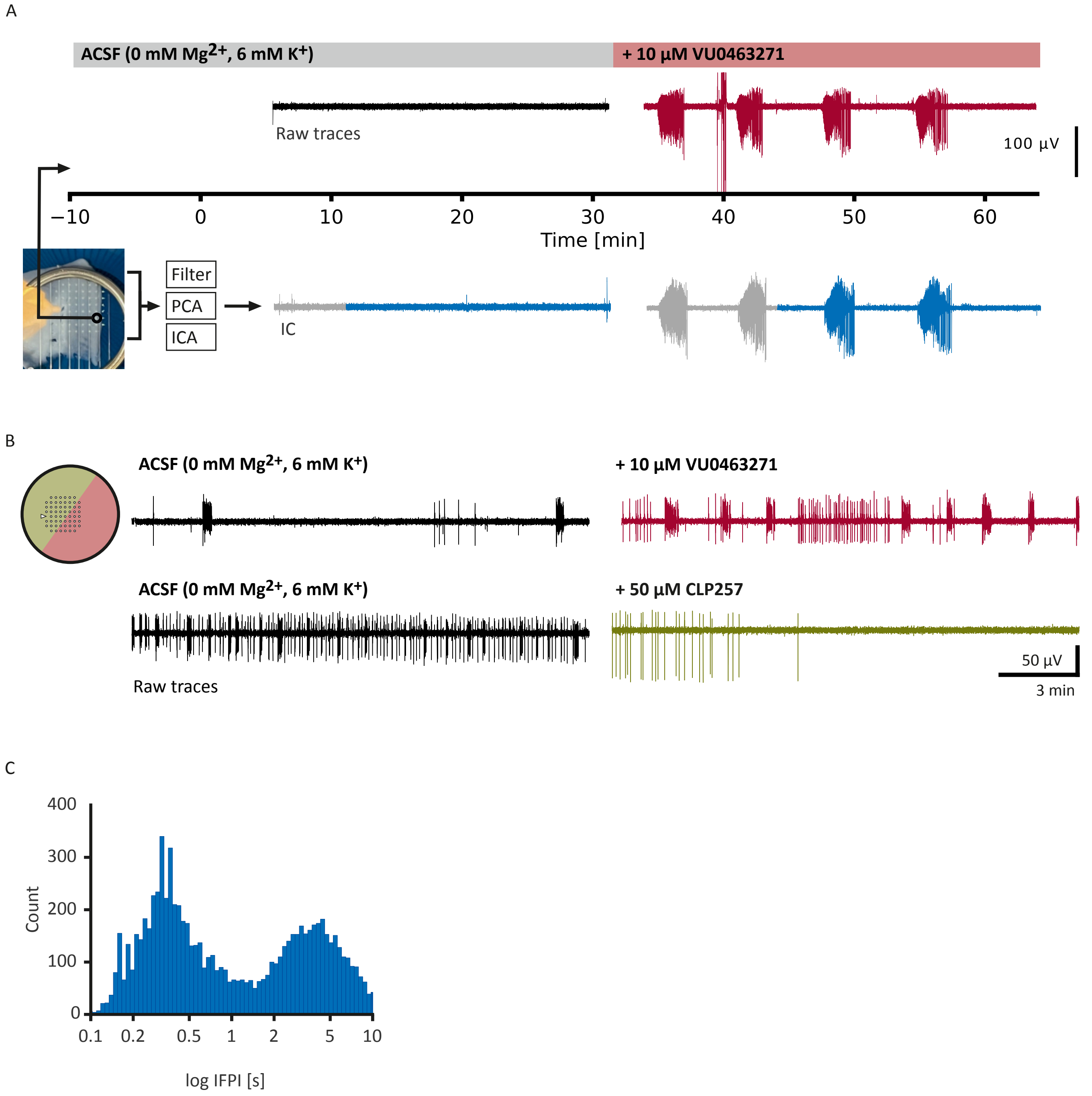

Figure S1: MEA analysis pipeline enables inter-field potential-interval based ILE detection under baseline and test conditions **(A)** Representative microelectrode array (MEA) recording and analysis workflow. *Upper panel:* Slices were perfused with modified ACSF (0 mM Mg^{2+}, 6 mM K^+, *grey* bar) for at least 22 min. Following 20 min of stable baseline recording, perfusion with 10 µM VU0463271 (*red bar*) was started, and recordings resumed once artifacts had subsided (2 min). *Lower panel:* Photograph of brain slices positioned on the electrodes and independent component (IC) trace of the source detected by our source separation pipeline. Minutes 12 to 32 of the IC under VU0463271 or respective substances and 20 min pre-wash-in were used for the analysis (*blue IC traces*). **(B)** Representative raw voltage traces from two patients with tumor-related temporal lobe epilepsy under baseline (*black*), VU0463271 (*red*), and CLP (*green*) conditions show comparable ILE features and KCC2-dependent effects as ILEs from TLE without tumors (e.g., in Figure 2A, 3B, S3A) **(C)** Inter-field potential-interval (IFIP) histogram of 20 IC traces with and without ILEs. The IFPI value in the bimodal distribution valley (1.5 s) was used as the maximum IFPI for ILE detection.

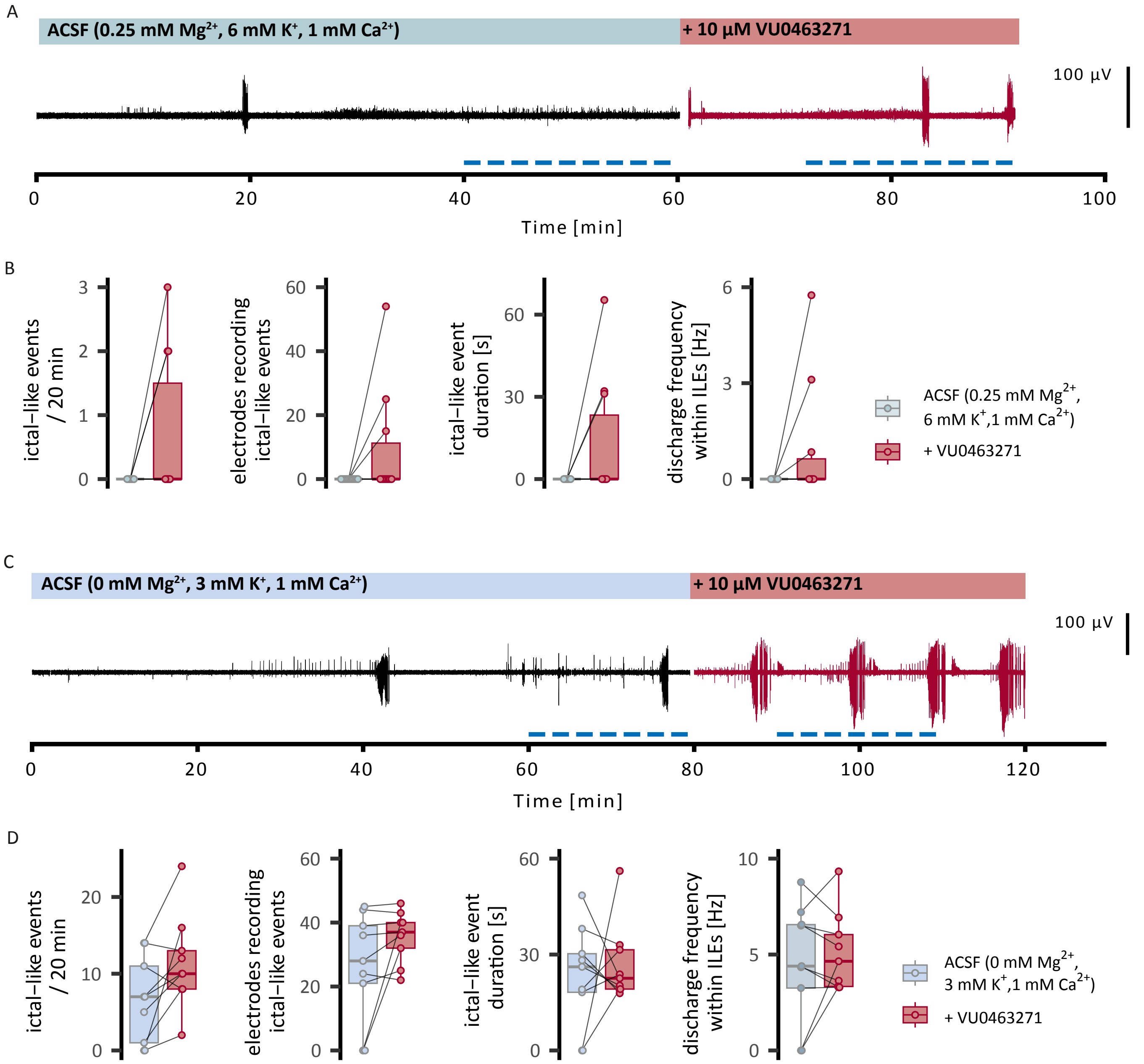

Figure S2: The effect of KCC2 blockade is not limited to ictal-like activity induced by 0 mM Mg^{2+} or elevated K^{+} **(A)** Representative raw voltage trace of ILEs induced by modified ACSF containing 0.25 mM Mg^{2+}, 6 mM K^{+}, and 1 mM Ca^{2+} before (*dark grey bar*) and during perfusion with 10 µM VU0463271 (*red bar*). We recorded a single ILE during 60 mins of control condition (in 1/10 slices, *N* = 3), and no ILEs in the 20 min before VU0463271 wash-in (hence in our analysis window, *blue dashed line*) **(B)** Population data on KCC2 blockade that led to (increased) ILE occurrence in 3 of 10 slices. ILEs were consistently observed in supragranular layers (3/3). KCC2 block led to an increase of interictal-like discharges (IILDs) in 4 of the 7 slices without ILEs (0.00 s^{-1}, interquartile range [IQR] = 3.3 to 0.04 s^{-1}, IQR = 2.7). **(C)** Representative raw voltage trace of ictal-like activity induced by modified ACSF containing 0 mM Mg^{2+}, 3 mM K^{+}, and 1 mM Ca^{2+} before (*light grey bar*) and during perfusion with 10 µM VU0463271 (*red bar*). **(D)** Population data (*n* = 9, *N* = 4) on VU0463271 induced increase in the number of ILEs (Δ: 5.2 ILEs / 20 min (95% credible interval [CrI]: 0.9;9.7, *descriptive statistics*: from 7, IQR = 10 to 10, IQR = 5) and an expansion of the spatial distribution of ILEs (Δ: 5.0 electrodes (95% CrI: –2.6;13.8, *descriptive statistics*: from 28 electrodes, IQR = 18 to 37 electrodes, IQR = 8). KCC2 inhibition did not significantly affect the duration of ILEs (*descriptive statistics*: from 26.2 s, IQR = 12.0 to 22.6 s, IQR = 12.3) nor the discharge frequency within ILEs (*descriptive statistics*: from 4.4 s^{-1}, IQR = 3.3 to 4.6 s^{-1}, IQR = 2.7). Under baseline conditions, ILEs were recorded in 7 of 9 slices. In this subset, ILEs had a mean number of 8.2 per 20 minutes (95% CrI: 3.2;13.1), a mean spatial distribution across 35 electrodes (95% CrI: 25.2;45.5), and a mean duration of 29.1 s (95% CrI: 17.9;40.2). The mean discharge frequency within ILEs was 5.7 s^{-1} (95% CrI: 3.4;8.2). ILEs consistently occurred in supragranular layers (7/7).

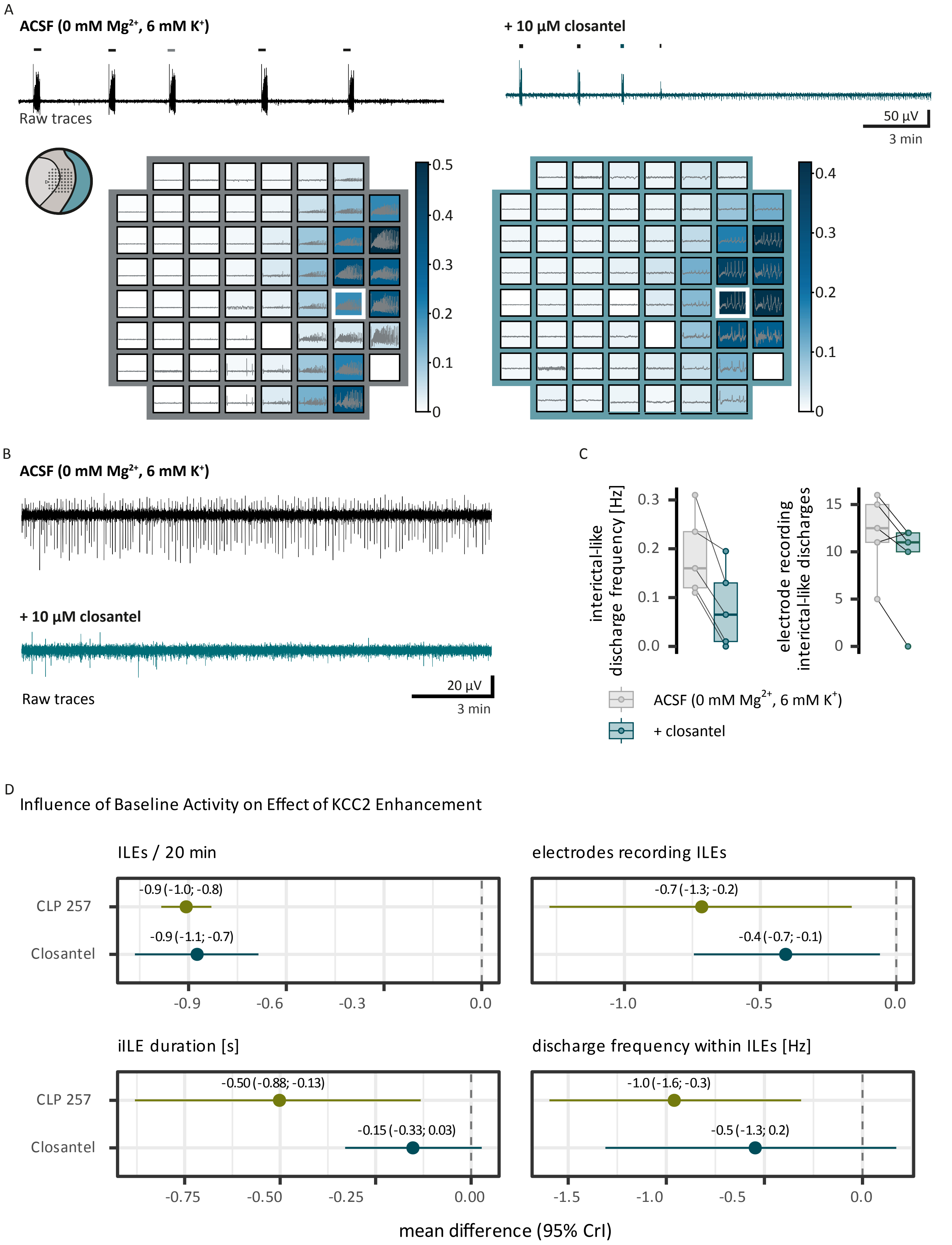

Figure S3: KCC2 enhancement via the WNK-SPAK pathway reduces the spatial distribution of induced ictal-like events (ILEs), and higher baseline activity increases the effects of the putative KCC2 enhancers CLP257 and closantel.

(A) Raw voltage traces and spatial heat maps of one neocortical brain slice before (*left*) and under (*right*) application of indirect KCC2 enhancer closantel. *Upper panel:* Raw traces before (*black*) and under 10 µM closantel (*teal*), recorded by the white-framed electrode. The bars on top of the traces indicate ILEs. *Lower panel:* Heat maps of ICA weights (ratio of the raw signal attributed to the IC of the source of ILEs) and traces of corresponding electrodes for the ILE highlighted above (*grey*/*teal*). Inset: slice position on electrode grid, the triangle represents the reference electrode. See Fig. 3C for population data. **(B)** Trace of induced inter-ictal like discharges (IILDs) before and under 10 µM closantel. **(C)** Closantel reduces the frequency of IILDs (*descriptive statistics*: from 0.16 s^{-1}, interquartile range [IQR] = 0.12 to 0.06 s^{-1}, IQR = 0.12, n = 5, N = 3), and the number of electrodes during IILDs (*descriptive statistics*: from 12.5 electrodes, IQR = 4.0 to 11.0 electrodes, IQR = 2.0, n = 5, N = 3). Because of the small sample size, inferential statistics were not performed. **(D)** Forest plots of the influence of baseline ILE activity on the effect size of KCC2 enhancement: The displayed coefficients with 95% credible intervals (CrI) indicate the change in enhancer effect for each increase in the baseline variable. Larger baseline values increased the effect of CLP257 and closantel on all outcome variables. Note that the effect of KCC2 enhancement is negative (Figure 3). A negative coefficient indicates an increase in the (negative) effect size.

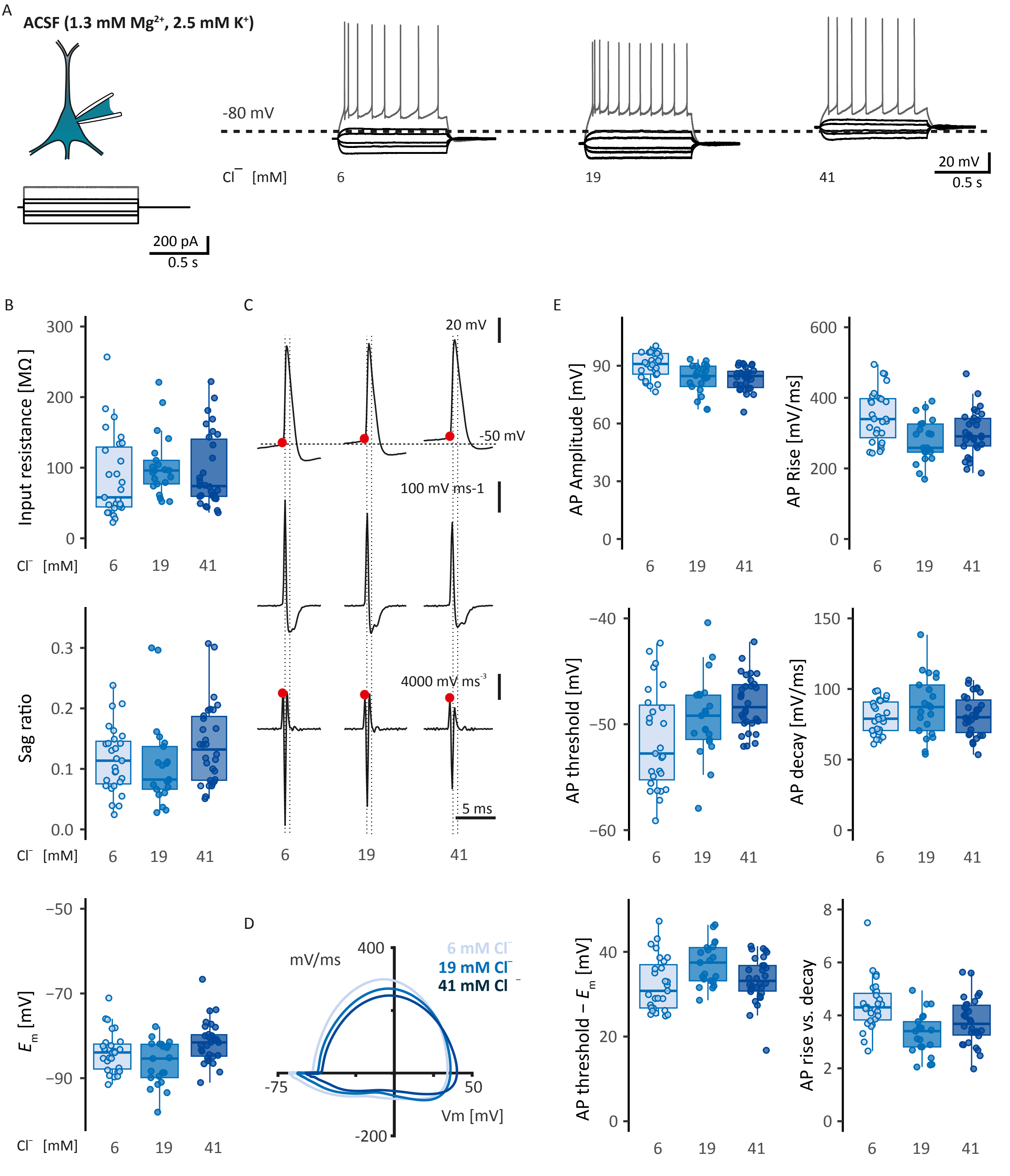

Figure S4: **Moderate $[Cl^-]_p$ deviations did not consistently alter intrinsic neuronal properties.** Altered $[Cl^-]_i$ might change the function of various ion channels (as for instance I_h[24]) and therewith potentially membrane properties. Although its generally recognized that altered Cl^- homeostasis *per se* does not change action potential properties, a massive $[Cl^-]_i$ overload in mouse hippocampal neurons lowered the threshold for action potential firing.[25] Based on this report, we systematically asked whether and how nominally defined somatic $[Cl^-]_i$ changes ($[Cl^-]_p$ = 6 – *light blue*, 19 – *blue*, or 41 mM – *dark blue*) influence intrinsic neuronal properties in temporal neocortical pyramidal neurons in the seizure propagation zone of humans suffering from TLE. Our aim was twofold; we firstly wanted to predict possible changes in membrane and firing properties that might occur with alterations of steady state $[Cl^-]_i$ upon KCC2 modulation[26] and may contribute (in addition to the known synaptic changes) to the network effect we report in the manuscript. Secondly, in case of clearly defined differences and correlation in specific parameters, we planned to use those parameters as a surrogate measure calibrated by the results depicted here. **(A)** *left*:

scheme of a somatic whole-cell recording. *Right:* Sub- (*black*) and supra- (*grey*) threshold voltage responses (*top*) to current injections of –200, –100, –50, 50 or 100 pA (*bottom*). In addition to the DIC appearance, attenuating AP frequency (here in response to 250 pA current injection) (*grey trace*) strongly supports pyramidal identity. **(B)** Population data of subthreshold membrane properties (6 mM: *n* = 27, *N* = 3, 19 mM: *n* = 21, *N* = 10, 41 mM: *n* = 30, *N* = 10). *Top:* input resistance (R_{in} from linear regression of the I-V relation at low current injections); *middle:* Sag ratio (difference between the most negative membrane potential during a hyperpolarizing current injection and steady-state voltage normalized to the maximum voltage response); *bottom*: Resting membrane potential (E_m). **(C)** Voltage trace (*upper row*) of action potentials (AP) characterized by AP threshold (red dots – first peak of the 3rd derivative of the voltage trace – *lower row*); AP rise /decay (most positive/negative peak (vertical dotted lines, respectively) of the 1st derivative of the voltage trace – middle row). **(D)** AP phase plot as a direct readout of net ionic current as a function of voltage. **(E)** Population data on AP characteristics. *Left top*: amplitude (threshold to peak); *left middle*: threshold; *left bottom*: distance of AP threshold from E_m as a measure of excitability; *right column*: AP kinetics. Minor changes in resting membrane potential and action potential properties did not seem to be directly linked to $[Cl^-]_i$ alterations, but rather represent compensating adaptations, given, for instance, the depolarized action potential threshold that was expected to be more hyperpolarized with increasing $[Cl^-]_i$. The variance between individual neurons and the very small (if any) effect at any given $[Cl^-]_p$ precludes the use of any of these parameters as a surrogate measure of $[Cl^-]_i$.[25]

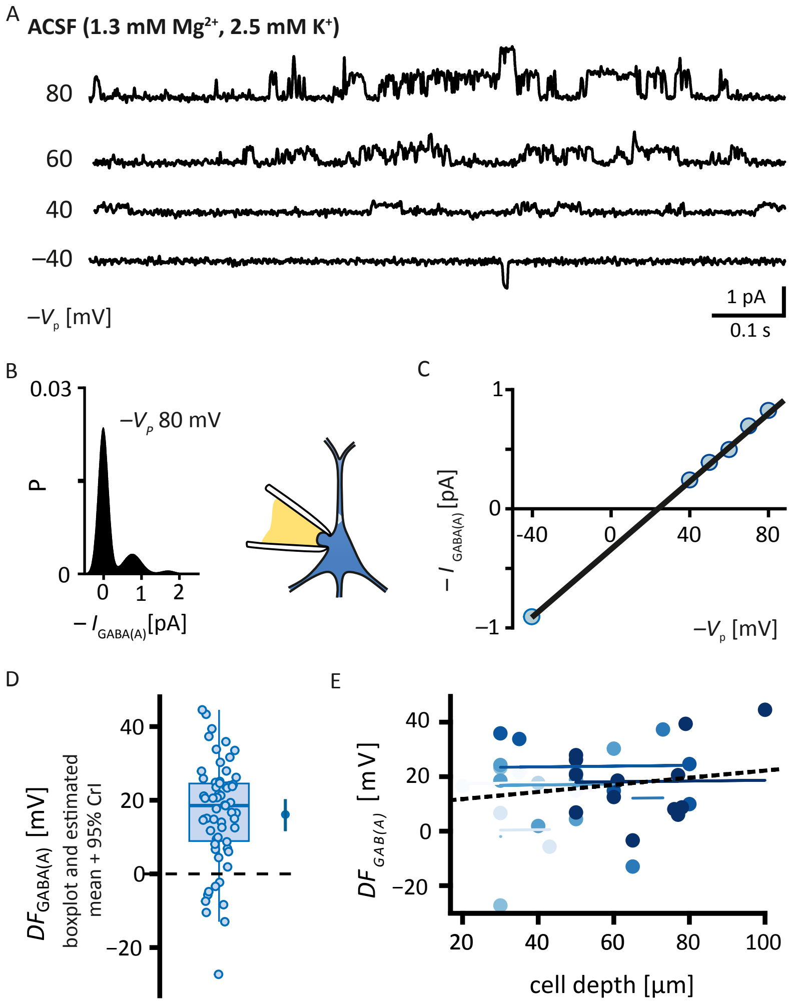

Figure S5: $GABA_A$ receptor driving force ($DF_{GABA(A)}$) in neocortical supragranular pyramidal neurons from the seizure propagation zone of patients with TLE favor a depolarizing anionic current in most neurons.

Because prominent autofluorescence in human neocortical neurons impeded a direct $[Cl^-]_i$ imaging approach, single channel currents were recorded in cell-attached configuration at different voltage levels for 10 s each (from –80 to 80 mV, increments 10 or 20 mV). Pipettes were pulled to have resistances of 7-10 MΩ when filled with pipette solution. Cell-attached single channel recordings are non-invasive, have a negligible effect on $E_{GABA(A)}$ or membrane potential (E_m),[27] and yield the $GABA_A$ receptor driving force ($DF_{GABA(A)}$ = inverted pipette potential (–V_p) when $GABA_A$ currents = 0). However, varying HCO_3^- levels or varying E_m might contribute to the measured findings. **(A)** Example of single channel $GABA_A$ currents at indicated inverted command voltages (–V_p) **(B)** Single channel current amplitudes extracted from fitted amplitude histograms exemplified at 80 mV (–V_p). *Inset:* Illustration of cell-attached configuration. **(C)** Corresponding IV plot of the example in (A) reveals a $DF_{GABA(A)}$ of 23.7 mV, and a conductance (G) of 14.4 pS.

(D) Population data of $DF_{GABA(A)}$, the estimated mean $DF_{GABA(A)}$ (adjusted for nested data) is plotted next to the boxplots. The estimated mean $DF_{GABA(A)}$ was 16.1 mV (95% CrI: 11.6; 20.3, n = 62, N = 12) (descriptive statistics: 18.5 mV, interquartile range [IQR] = 15.6, positive $DF_{GABA(A)}$ in 86 %, negative in 14 %), estimated mean $G_{GABA(A)}$ was 17.5 pS (95% CrI: 15.3; 19.8) (*descriptive statistics:* 15.6 pS, IQR = 4.6). Note that the permeability for HCO_3- shifts $E_{GABA(A)}$ by 10 – 15 mV in a positive direction depending on pH_i[28] but the influence on $DF_{GABA(A)}$ might be less prominent due to the concomitant depolarization of the membrane.
Since we cannot exclude a major contribution of perturbed Cl^- homeostasis in a subset of neurons as shown for glioma cells,[11] we separately analyzed $DF_{GABA(A)}$ and $G_{GABA(A)}$ in tumor-related TLE versus TLE patients without tumors and found no difference (Δ $DF_{GABA(A)}$TLE – $DF_{GABA(A)}$ tumor-related TLE: 2.06 mV (95% CrI: –7.2; 11.2, n = 62, N = 12), Δ $G_{GABA(A)}$ TLE – $G_{GABA(A)}$ tumor-related TLE: – 3.6 pS (95% CrI: –8.5; 1.3, n = 62, N = 12)). **(E)** Neurons were recorded between 20 and 100 µm depth in the slice. $DF_{GABA(A)}$ and cell depth showed no correlation (repeated measures correlation: r = 0.01, df = 26, 95% confidence interval (CI): – 0.36; 0.39, n = 35, N = 8). Each patient is represented by a different color. Parallel lines are fitted for each patient, with the dashed black line representing the overall regression. Note, that the percentage of $DF_{GABA(A)}$ variance explained by the variance between patients was 4.3 % (ICC from mixed effects model), indicating that most of the variability is at a cellular rather than interindividual level. In conclusion, a higher proportion of neurons in neocortical brain slices from the seizure propagation zone of our TLE patients had depolarizing somatic $DF_{GABA(A)}$ compared to previously reported human neurons (for hippocampal TLE neurons: 20% positive $DF_{GABA(A)}$, E_m = –61.8 mV, for neocortical TLE neurons: E_m = –53.7 mV; 50 % $E_{GABA(A)}$ = –55.2 mV, 50% $E_{GABA(A)}$ = –68.9 mV).[29,30] This may partially be attributed to the strict somatic recordings and – although not recorded at the given neurons – the on average more hyperpolarized membrane potential of layer 2/3 neocortical neurons (Figure S4) compared to hippocampal neurons. Please note that these results from cell-attached recordings might serve as a hint, but Cl^- extrusion capacity cannot be reliably estimated when measuring E_{GABA} ($DF_{GABA(A)}$) or even $[Cl^-]_i$ in resting neurons (in the absence of Cl-loading) as explained by the load pump paradigm and demonstrated experimentally [31]

A

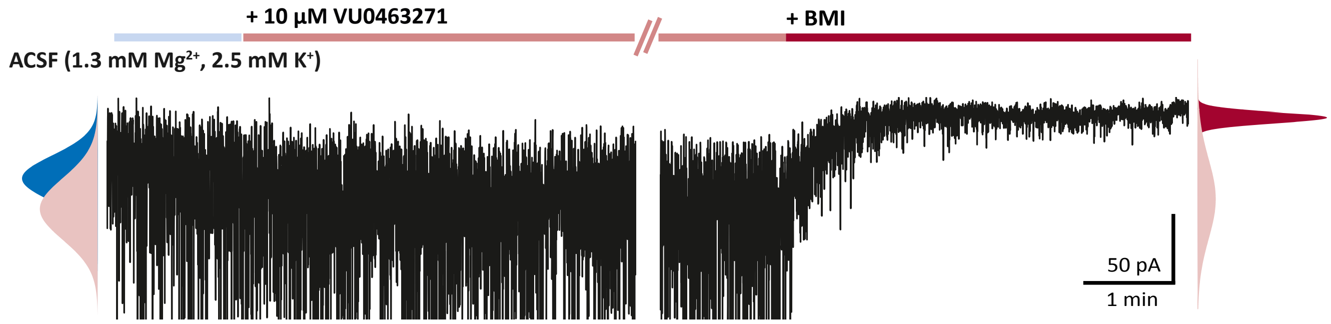

B

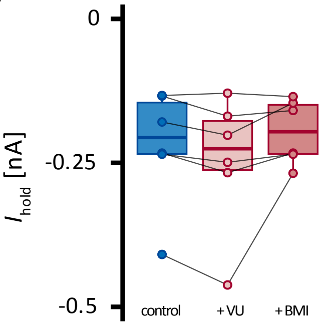

Figure S6: Preliminary data on the influence of acute VU0463271 application on tonic inhibition. (A) Somatically recorded current trace from a supragranular neocortical pyramidal neuron filled with a CsCl-based solution and voltage clamped at –73 mV in the presence of glutamate receptor blockers CNQX (20 µM), and DAP5 (25 µM) at baseline (*light blue bar*) during the application of KCC2 blocker VU0463271 (10 µM, *pink bar*) and subsequent additional application of GABA(A) receptor blocker bicuculline (BMI, 10 µM, *red bar*). Large phasic events were cut for clarity. All-point current histogram peaks of baseline (*light blue*), during VU0463271 application (10 min *pink*), and during VU0463271 and BMI application (*red*) were taken to estimate the actual holding current. Diagonal lines denote break in continuous recording. **(B)** KCC2 block (*middle, pink*) decreased I_{hold} (*left, light blue*) from –206.0 pA, interquartile range [IQR] = 89.2 to –225.5 pA, IQR = 85.2. Application of BMI (*right, red*) increased $I_{hold\text{-}VU0463271}$ to –196.0 pA, IQR = 85.2, resulting in a VU0463271-mediated tonic inhibition of 24.0 pA, IQR = 39.5 (*descriptive statistics, n* = 6, *N* = 1*).*

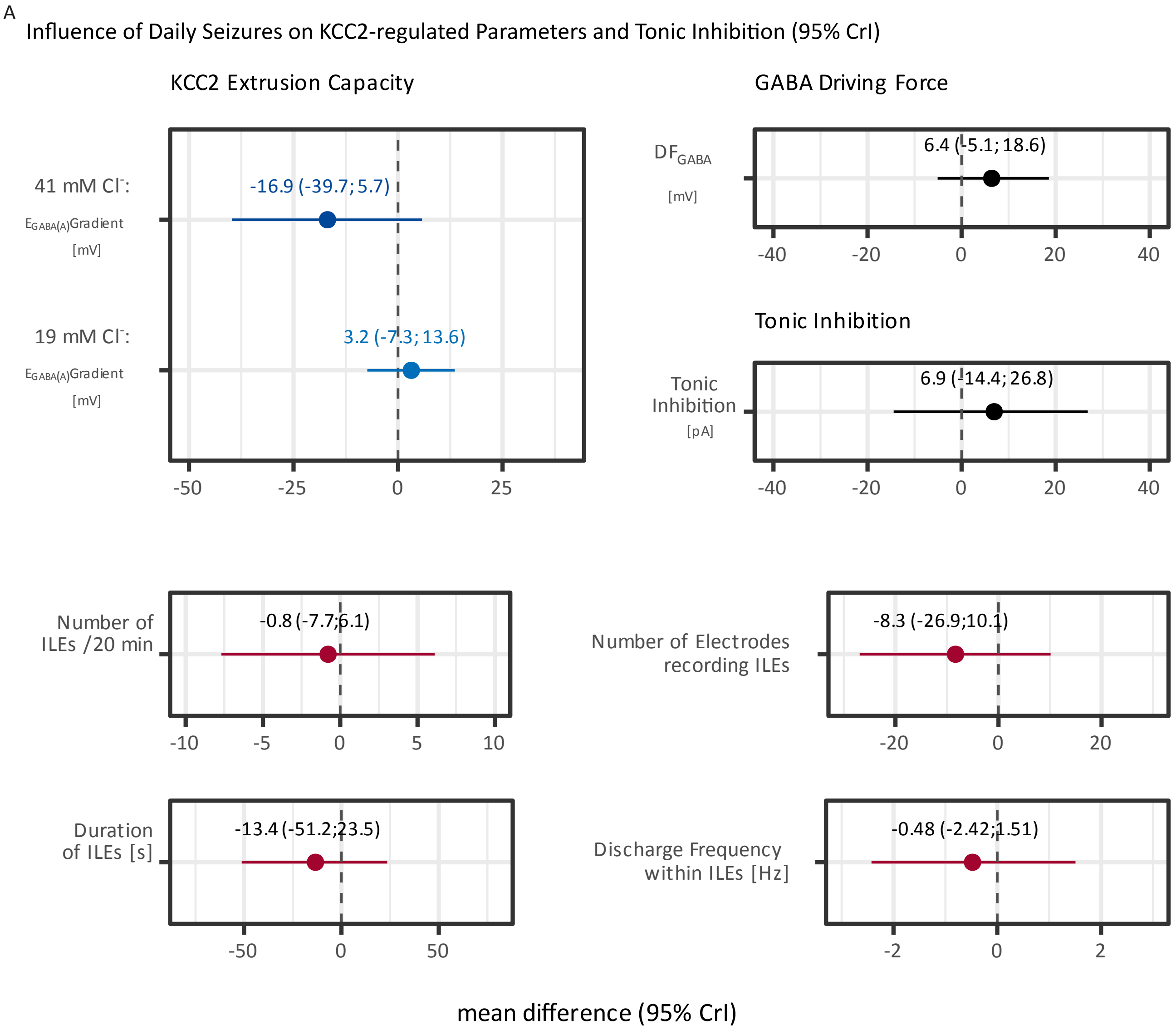

Figure S7: Preoperative seizure frequency putatively decreases KCC2 function and increases tonic inhibition in the seizure propagation zone of neocortical brain tissue of TLE patients. Displayed coefficients with 95% credible intervals (CrI) describe the effect of daily seizures on outcome variables. **(A)** *Left panel:* Daily seizures were associated with a decrease in the $E_{GABA(A)}$ gradient at 41 mM $[Cl^-]_p$, but not at 19 mM $[Cl^-]_p$. *Right panel:* Daily seizures were associated with a slightly increased $DF_{GABA(A)}$ and tonic inhibition. **(B)** In line with the tendencies of the extrusion capacity at 41 mM $[Cl^-]_p$, daily seizures were associated with a decreased VU0463271 effect on the spatial distribution. However, daily seizures negligibly affected the impact of the KCC2 blocker on the number of ILEs / 20min, duration of ILEs, and discharge frequency within ILEs.

Supplementary References

1. Pedregosa, F. *et al.* Scikit-learn: Machine Learning in Python. *Journal of Machine Learning Research* **12**, 2825–2830 (2011).
2. Rivera, C. *et al.* Mechanism of activity-dependent downregulation of the neuron-specific K-Cl cotransporter KCC2. *Journal of Neuroscience* **24**, 4683–4691 (2004).
3. Lee, H. H. C., Deeb, T. Z., Walker, J. A., Davies, P. A. & Moss, S. J. NMDA receptor activity downregulates KCC2 resulting in depolarizing GABAA receptor-mediated currents. *Nat Neurosci* **14**, 736–743 (2011).
4. Puskarjov, M., Ahmad, F., Kaila, K. & Blaesse, P. Activity-dependent cleavage of the K-Cl cotransporter KCC2 mediated by calcium-activated protease calpain. *Journal of Neuroscience* **32**, 11356–11364 (2012).
5. Rivera, C. *et al.* BDNF-induced TrkB activation down-regulates the K+-Cl- cotransporter KCC2 and impairs neuronal Cl- extrusion. *Journal of Cell Biology* **159**, 747–752 (2002).
6. Wake, H. *et al.* Early changes in KCC2 phosphorylation in response to neuronal stress result in functional downregulation. *Journal of Neuroscience* **27**, 1642–1650 (2007).
7. Burman, R. J. *et al.* Excitatory GABAergic signalling is associated with benzodiazepine resistance in status epilepticus. *Brain* **142**, 3482–3501 (2019).
8. Huberfeld, G. *et al.* Glutamatergic pre-ictal discharges emerge at the transition to seizure in human epilepsy. *Nat Neurosci* **14**, 627–634 (2011).
9. Kurki, S. N. *et al.* Expression patterns of NKCC1 in neurons and non-neuronal cells during cortico-hippocampal development. *Cereb Cortex* **33**, 5906–5923 (2023).
10. Conti, L. *et al.* Anomalous levels of Cl- transporters cause a decrease of GABAergic inhibition in human peritumoral epileptic cortex. *Epilepsia* **52**, 1635–1644 (2011).
11. Pallud, J. *et al.* Cortical GABAergic excitation contributes to epileptic activities around human glioma. *Sci Transl Med* **6**, 244ra89 (2014).
12. Virtanen, M. A., Uvarov, P., Mavrovic, M., Poncer, J. C. & Kaila, K. The Multifaceted Roles of KCC2 in Cortical Development. *Trends Neurosci* **44**, 378–392 (2021).
13. Puskarjov, M., Kahle, K. T., Ruusuvuori, E. & Kaila, K. Pharmacotherapeutic targeting of cation-chloride cotransporters in neonatal seizures. *Epilepsia* **55**, 806–18 (2014).
14. Nguyen, T. D. *et al.* Astrocytic NKCC1 inhibits seizures by buffering Cl− and antagonizing neuronal NKCC1 at GABAergic synapses. *Epilepsia* **64**, 3389–3403 (2023).
15. Gelman, A. & Hill, J. *Data Analysis Using Regression and Multilevel/Hierarchical Models*. *Data Analysis Using Regression and Multilevel/Hierarchical Models* (Cambridge University Press, 2006). doi:10.1017/CBO9780511790942.
16. Murtaugh, P. A. Simplicity and complexity in ecological data analysis. *Ecology* **88**, 56–62 (2007).
17. Barr, D. J., Levy, R., Scheepers, C. & Tily, H. J. Random effects structure for confirmatory hypothesis testing: Keep it maximal. *J Mem Lang* **68**, 255–278 (2013).
18. R Core Team (2025). _R: A Language and Environment for Statistical Computing. Preprint at <https://www.R-project.org/> (2025).
19. Bürkner, P. C. brms: An R package for Bayesian multilevel models using Stan. *J Stat Softw* **80**, (2017).
20. Bakdash, J. Z. & Marusich, L. R. Repeated Measures Correlation. *Front Psychol* **8**, 456 (2017).
21. Wickham, H. *Ggplot2: Elegant Graphics for Data Analysis*. *Journal of Statistical Software* vol. 35 (Springer-Verlag New York, New Yorck, 2016).
22. Pedersen, T. L. patchwork: The Composer of Plots. Preprint at https://cran.r-project.org/package=patchwork (2024).
23. Makowski, D., Ben-Shachar, M. & Lüdecke, D. bayestestR: Describing Effects and their Uncertainty, Existence and Significance within the Bayesian Framework. *J Open Source Softw* **4**, 1541 (2019).

24. Mistrík, P., Pfeifer, A. & Biel, M. The enhancement of HCN channel instantaneous current facilitated by slow deactivation is regulated by intracellular chloride concentration. *Pflugers Arch* **452**, 718–727 (2006).

25. Sørensen, A. T. *et al.* Altered Chloride Homeostasis Decreases the Action Potential Threshold and Increases Hyperexcitability in Hippocampal Neurons. *eNeuro* **4**, 1–10 (2017).

26. Doyon, N. *et al.* Efficacy of synaptic inhibition depends on multiple, dynamically interacting mechanisms implicated in chloride homeostasis. *PLoS Comput Biol* **7**, e1002149 (2011).

27. Tyzio, R. *et al.* Postnatal changes in somatic γ-aminobutyric acid signalling in the rat hippocampus. *European Journal of Neuroscience* **27**, 2515–2528 (2008).

28. Kaila, K. Ionic basis of GABAA receptor channel function in the nervous system. *Prog Neurobiol* **42**, 489–537 (1994).

29. Huberfeld, G. *et al.* Perturbed chloride homeostasis and GABAergic signaling in human temporal lobe epilepsy. *Journal of Neuroscience* **27**, 9866–9873 (2007).

30. Deisz, R. A., Lehmann, T. N., Horn, P., Dehnicke, C. & Nitsch, R. Components of neuronal chloride transport in rat and human neocortex. *Journal of Physiology* **589**, 1317–1347 (2011).

31. Jin, X., Huguenard, J. R. & Prince, D. A. Impaired Cl- extrusion in layer V pyramidal neurons of chronically injured epileptogenic neocortex. *J Neurophysiol* **93**, 2117–2126 (2005).
